# Host–guest complexation of progesterone with β-cyclodextrin derivatives: hydration structure, binding thermodynamics, and loading pathways from molecular dynamics simulations

**DOI:** 10.1039/d6ra04566a

**Published:** 2026-07-30

**Authors:** Faezeh Mobini, Marina Provenzano, Nada Alghamdi, Matteo Fasano, Mokhtar Ganjali Koli

**Affiliations:** a Venom and Biotherapeutics Molecules Lab, Medical Biotechnology Department, Biotechnology Research Center, Pasteur Institute of Iran Tehran Iran; b Department of Energy, Politecnico di Torino Corso Duca degli Abruzzi 24 10129 Torino Italy; c Department of Chemistry, University of Kurdistan Sanandaj Iran m.ganjalikoli1360@gmail.com; d Computational Chemistry Laboratory, Kask Afrand Exire Ltd Sanandaj Iran

## Abstract

Progesterone (PROG) is a poorly water-soluble steroid whose formulation remains challenging despite the broad pharmaceutical utility of cyclodextrins (CDs). Here, atomistic molecular dynamics simulations combined with umbrella sampling and potential of mean force (PMF) reconstruction were used to compare the inclusion behavior of PROG in native β-cyclodextrin (βCD) and three pharmaceutically relevant R_2_-substituted derivatives, namely 2-methyl-βCD, 2-hydroxypropyl-βCD, and 2-sulfobutylether-βCD. Twenty independent host–guest systems were constructed to characterize loading pathways, structural adaptation of the host cavity, hydration reorganization, and thermodynamic preference. The results show that R_2_ functionalization strongly modulates both directional selectivity of guest entry and the free-energy landscape of encapsulation. PROG binding induces a systematic reorganization of the cyclodextrin scaffold toward thicker, more circular, and more symmetric toroidal conformations, while partially dehydrating the cavity and perturbing the local tetrahedral structure of confined water. PMF analysis revealed pathway-dependent differences in the depths and shapes of the free-energy wells along the selected dissociation coordinate. Among the investigated pathways, particularly deep PMF minima were observed for specific loading pathways of 2-hydroxypropyl-βCD and 2-sulfobutylether-βCD. Across all carriers, van der Waals interactions dominate the direct short-range PROG–CD interaction, whereas short-range interactions between the PROG–CD complexes and surrounding water are strongly dependent on substituent chemistry. In particular, 2-sulfobutylether-βCD exhibits markedly more negative short-range complex–water interaction energies, primarily because of the electrostatic interactions between its sulfonate groups and surrounding water. These findings provide molecular-level insight into how cyclodextrin functionalization governs progesterone encapsulation and may guide the rational design of CD-based delivery systems.

## Abstract 2

Molecular dynamics simulations reveal how β-cyclodextrin derivatives modulate progesterone loading through cavity dehydration, host reorganization, and pathway-dependent binding thermodynamics.

## Graphical abstract

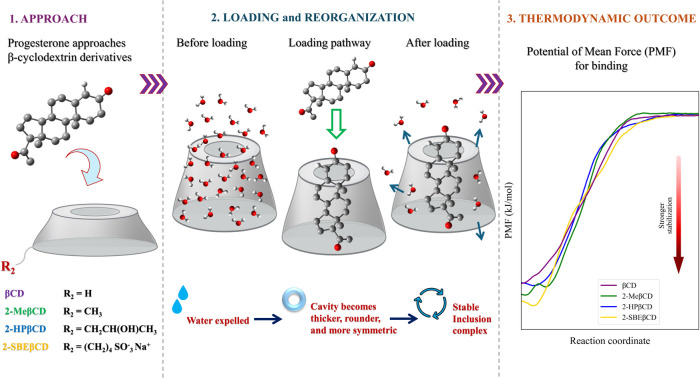

## 1 Introduction

Progesterone (PROG), a key steroid sex hormone, plays a crucial role in the regulation of various physiological processes, including reproductive function, maintenance of pregnancy, modulation of the immune response, and the treatment of various acute or chronic gynecological conditions.^1,2^ When endogenous progesterone production is insufficient, PROG can be administered *via* different routes such as oral, vaginal, transdermal, topical, parental, and intranasal routes. Although PROG is commercially available in multiple conventional formulations, low solubility, limited permeability and extensive hepatic first-pass metabolism are the major constraints to its delivery.^3^

CDs are cyclic oligosaccharides composed of glucopyranose units able to enhance the solubility and stability of some hydrophobic guest molecules. They are produced from starch using hydrolytic enzymes and possess a hydrophobic cavity and a hydrophilic surface, making them valuable for applications in pharmaceuticals, biotechnology, environmental science, and materials engineering.^4–6^ The ability of CDs to increase the aqueous solubility and bioavailability of some poorly soluble drugs such as PROG,^7^ while at the same time removing the same compound as an environmental pollutant,^8^ indicates the high potential of these compounds for multipurpose applications.

Recent advances in CD-based drug delivery include CD nanosponges (CDNSs), which have shown promising potential for enhancing the aqueous solubility and bioavailability of poorly water-soluble compounds, including curcumin, resveratrol, oxyresveratrol, and quercetin.^9^

Previous experimental studies have demonstrated improved encapsulation efficiency and release behavior of progesterone in β-cyclodextrin-based systems, with successful complex formation confirmed at a 1 : 1 molar ratio and enhanced release profiles compared to the free drug.^10^ Although experimental studies provide valuable insights into PROG–CD interactions, they primarily offer macroscopic observations of binding affinity and solubility. However, they do not capture atomic-level interactions, conformational dynamics, and detailed thermodynamic stability. Molecular dynamics (MD) simulations can bridge this gap by offering time-resolved insights into the binding mechanisms, stability, and energetics of drug–CD complexes.^11–13^

Fereidounpour *et al.* utilized MD simulations to gain crucial understanding about the interactions between steroids and CDs.^14^ According to their results, among the three native CDs (α-, β-, and γ-CDs), βCD forms the most stable inclusion complexes with dexamethasone (−21.23 kJ mol^−1^) and hydrocortisone (−20.09 kJ mol^−1^). These results underscore the significance of cavity size and ligand orientation in binding stability. The simulations were able to model the conformational changes of steroid–CD complexes, providing important molecular-level insights into complex stability and the mechanisms governing their formation.

Mazurek *et al.* demonstrated that the inclusion complex of 17-β-estradiol (EST) with βCD has a stable 1 : 2 stoichiometry.^15^ MD simulations demonstrated the dynamic stability of this complex, characterized by a preferred orientation where the steroidal phenolic ring of EST resides near the narrow rim of βCD. The binding free energy calculations (Δ*G*_bind_ = −13.66 ± 4.20 kcal mol^−1^ for the 1 : 2 complex) were consistent with experimental Gibbs free energy (Δ*G* = −9.92 kcal mol^−1^). These results indicated that the 1 : 2 stoichiometry is energetically and dynamically favored over the previously hypothesized 1 : 1 ratio.

**Fig. 1:**
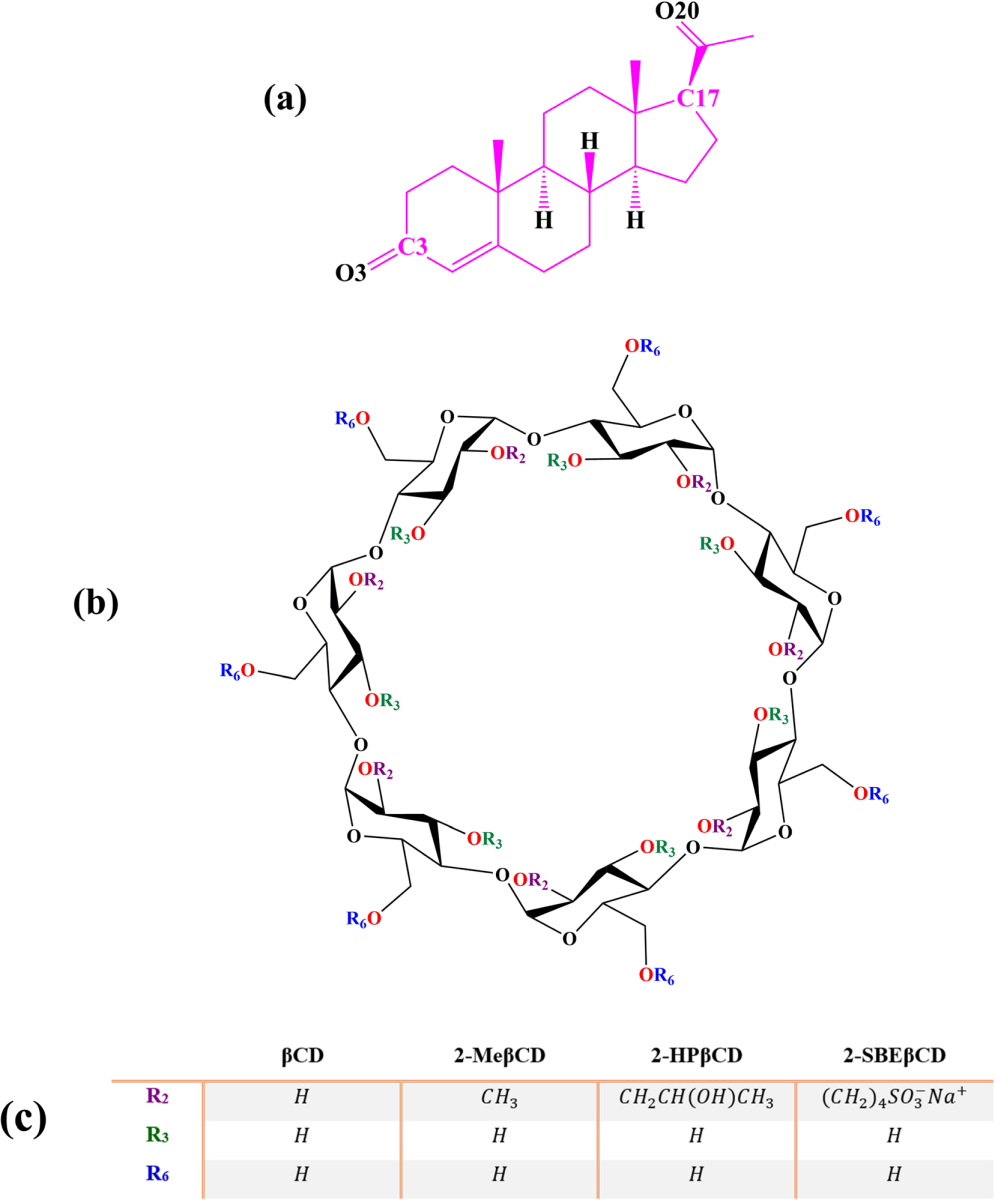
Chemical structures of (a) progesterone, (b) the main skeleton of native βCD, and (c) its chemically modified derivatives.

On the other hand, PROG consists of a core structure composed of four fused carbon rings (cyclopentanoperhydrophenanthrene), as shown in Fig. 1a. This rigid, nonpolar backbone limits interaction with water and enhances hydrophobicity.^16^ Its high partition coefficient (Log *P* = 3.9) indicates strong lipophilic behavior and a tendency to dissolve in lipid environments rather than aqueous solutions, resulting in a very low aqueous solubility of 0.007–0.0168 mg mL^−1^.^17–21^ Additionally, it interacts with biological receptors and serum proteins primarily *via* hydrophobic forces, as seen in its binding to PROG receptors and serum albumin 22. Thus, PROG is labeled as “practically insoluble”, according to the solubility classification adopted by USP and Ph. Eur, and is classified as Class II drug (poor solubility high permeability) by the Biopharmaceutical Classification System (BCS).^23^

Given these safety concerns, this study aims to investigate the potential of non-toxic βCD derivatives as alternative carriers for PROG. However, a comprehensive comparative analysis of how functional substitutions at the R_2_ position of βCD influence the loading mechanisms and thermodynamic stability of progesterone inclusion complexes remains largely unexplored. Therefore, this work provides a molecular-level investigation of PROG inclusion in several pharmaceutically relevant βCD derivatives, including 2-methyl-βCD (2-MeβCD), 2-hydroxypropyl-βCD (2-HPβCD), and 2-sulfobutylether-βCD (2-SBEβCD), in which the functional substitutions at the R_2_ position are illustrated in Fig. 1b, c and S1, using molecular dynamics simulations combined with PMF-based free-energy calculations.

## 2 Computational details

### 2.1 Simulation setup

In the first step, the native βCD structure was selected as the reference. To sample different approach configurations, one PROG molecule was placed in five predefined orientations around βCD (1 : 1 ratio), with the closest interatomic distance set to approximately 1.6 nm (Fig. S2). Each orientation was simulated as an independent replicate. In the next step, the R_2_ position of the βCD ring was functionalized with different functional groups (Fig. 1c), resulting in the preparation of three modified carriers. The same five initial PROG orientations were used for each carrier. Consequently, four types of carriers (one native and three functionalized) were evaluated in interaction with PROG. In total, 20 PROG-CD systems were designed, along with separate simulations of each carrier and PROG in water.

Each system was explicitly solvated with 4500 water molecules. This number was chosen to provide a sufficiently dilute host–guest system under bulk aqueous conditions. In periodic MD simulations, the solvent content should be sufficient to minimize artefacts arising from interactions between the solute and its periodic images, while avoiding unnecessarily large simulation boxes that provide limited additional benefit at substantially increased computational cost.^24,25^ The same number of water molecules was used for all systems to ensure consistent solvent conditions and enable direct comparison among the different CD carriers.

### 2.2 MD simulation details

The molecular dynamics (MD) simulations were performed using GROMACS 2022.2 (ref. 26–28) with the GROMOS 54a7 force field.^29,30^ Water molecules were modeled using the SPC/E model.^31,32^ In systems containing 2-SBEβCD, seven Na^+^ ions were added for charge neutrality. Periodic boundary conditions were applied in all directions. Energy minimization was conducted using the steepest descent algorithm,^33^ followed by equilibration in the NVT and NPT ensembles for 1 ns and 9 ns, respectively. Production simulations were performed for 200 ns for each system, except for two replicas that were extended to 500 ns to complete the loading process. The systems were simulated with the leap-frog integrator, using a time step of 2 fs.^34^ Bond constraints were applied using the LINCS algorithm.^35^ van der Waals (vdW) interactions were truncated at 1.2 nm, and long-range electrostatics were treated using the Particle Mesh Ewald (PME) method^36^ with the same cutoff. Temperature was controlled at 300 K using the V-rescale thermostat (*τ* = 0.1 ps),^37^ and pressure was maintained at 1 bar using the Parrinello–Rahman barostat (*τ* = 2.0 ps).^38^ Hydrogen bonds were identified using a donor–acceptor distance cutoff of 0.35 nm and a hydrogen–donor–acceptor angular cutoff of 30°, where an angle of 0° corresponds to a fully extended hydrogen-bond geometry.

### 2.3 Potential of mean force

To characterize the pathway-dependent free-energy profiles associated with PROG displacement from the CD cavity, potential of mean force (PMF) calculations were performed using umbrella sampling.^39,40^ Final host–guest configurations obtained from the last frame of the production MD simulations, where PROG was fully accommodated inside the CD cavity, were used as initial structures. Each system was equilibrated under restrained conditions for 1 ns (NVT) followed by 9 ns (NPT), with positional restraints applied to the CD O1 atoms (Fig. S1) and all PROG atoms. Pulling simulations were performed along the principal axis of the CD cavity (*z*-direction) using a harmonic potential with a force constant of 500 kJ mol^−1^ nm^−2^ and a pulling rate of 0.005 nm ps^−1^, over a distance of ∼2.5 nm. This generated 24 umbrella sampling windows spaced at ∼0.1 nm intervals. Each window was equilibrated for 1 ns under NPT conditions with positional restraints, followed by a 10 ns production run. The first 5 ns were discarded, and the remaining data were used to construct PMF profiles using the WHAM method^41,42^ with 200 bins and 200 bootstrap iterations. Additional methodological details, including the definition of reference atoms, initial configuration schemes, and extended simulation protocols for selected replicas, are provided in Section S1 of the SI.

## 3 Results and discussions

### 3.1 Equilibrium distances and loading pathway

**Fig. 2:**
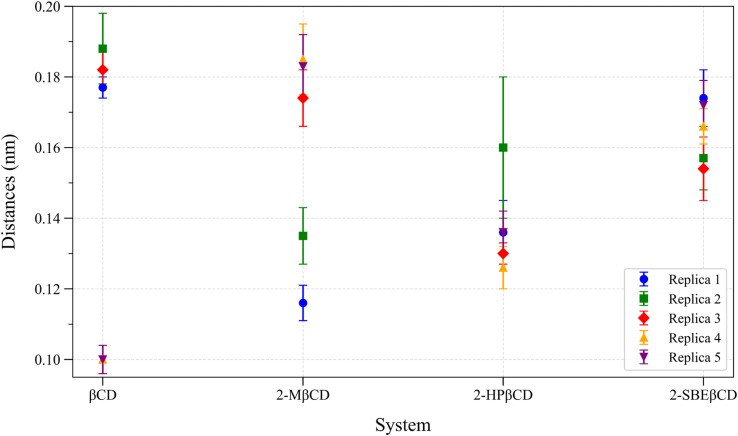
Comparison of the equilibrium distance between the COG of PROG and the centers of CD cavities across different replicas. Error bars indicate the statistical uncertainties estimated from block averaging.

Comprehending the loading mechanism of drugs into CDs (such as their orientation, depth, and interaction forces) is essential for the creation of effective inclusion complexes. This mechanistic understanding not only affects solubility, release rate, stability, and the encapsulation efficiency, but also influences the CD's effectiveness in masking unpleasant tastes or smells and minimizing irritation or toxicity.^43,44^ Therefore, the distances between the centers of geometry (COG) of PROG and the CD molecules were analyzed over time. The time evolution of these distances is provided in Fig. S3, while the equilibrium distances were calculated from the final 10% of the simulation time and are summarized in Fig. 2.

Generally, PROG approached the CD cavity, followed by reorientation and eventual insertion into the host structure, leading to a stable inclusion complex. Representative snapshots of the loading process, including the entry and final states of PROG for different replicas, are shown in the SI (Fig. S4–S7).

For the native βCD system, PROG successfully entered the cavity and reached a stable equilibrium position, as reflected by the stabilization of the host–guest distance over time. A similar loading mechanism was observed for the functionalized CDs, although differences in the equilibration behavior and stability of the complexes were evident. In the case of 2-MeβCD, PROG achieved a stable bound state with fluctuations comparable to those observed for βCD. For 2-HPβCD, the loading process exhibited a relatively slower equilibration in some cases, while still converging to a stable inclusion configuration. In contrast, 2-SBEβCD showed a more stable binding behavior, with reduced fluctuations around the equilibrium position, suggesting a more confined and stable bound state. The energetic basis for this stability is discussed in detail in Sections 3.4.1 and 3.4.2.

These results demonstrate that functionalization of βCD influences the observed loading behavior and the structural stability of the resulting complexes, while preserving the general inclusion mechanism.

### 3.2 Structural characteristics of CDs

The structural parameters of CDs, such as cavity height, circularity, and geometric anisotropy, play a crucial role in determining the specificity of molecular recognition and the thermodynamic stability of inclusion complexes. These parameters directly influence the enthalpic and entropic contributions to host–guest binding interactions. The stability, selectivity, and solubility of CD-drug complexes are directly impacted by these structural characteristics, making CDs valuable tools in molecular encapsulation, recognition, and controlled release applications.^45–47^

#### 3.2.1 Shape descriptors

The structural shape descriptors (*κ*^2^, *b*, and *c*)^48,49^ exhibited very small replica-to-replica variations. For all systems, the relative deviations between replicas were below ∼5%, confirming the consistency and reproducibility of the simulations. Therefore, the average values were reported, as seen in Fig. S8. This indicates that PROG behaves similarly in all directions after being placed inside the cavity of each CD, regardless of the loading mechanism.

Examination of geometric quantities extracted from the gyration tensor shows that the presence of PROG induces distinctive patterns in the shape and symmetry of CDs. In βCD, *b* increases by approximately 7–8%, while *c* decreases by about 2–3%, indicating a slight tendency toward axial elongation accompanied by a more circular cross-section. At the same time, *κ*^2^ increases by only about 1%, indicating that the overall anisotropy remains essentially unchanged and that the observed variations are primarily local geometric adjustments rather than global shape changes.

In the substituted derivatives, the pattern is reversed. A significant decrease in *b* indicates a weakening of axial elongation and a shift toward more isotropic symmetry, and this trend is corroborated by a substantial decrease in *κ*^2^, *i.e.*, a more spherical structure on a global scale. In addition, an increase in *c* indicates that in the cross-section, the mass distribution between the two minor axes becomes less uniform and local ellipticity/anisotropy is enhanced. The intensity of this effect varies among the derivatives, with 2-SBEβCD showing the most pronounced decrease in *κ*^2^, indicative of the greatest increase in global symmetry. Detailed definitions and the corresponding descriptor values are provided in the Section S3.

#### 3.2.2 Circularity

The circularity of the CDs rims, which comprise O_2_ and O_6_ atoms, is defined as the ratio of the smallest distance to the largest distance between any two adjacent O_2_ (and O_6_) atoms within the rims.^50^ We consider circularity to be a measure of the roundness of the cavity rims (PHR and SHR), ranging from 0 to 1: the closer the value is to 1, the more uniform and circular the rim geometry; the smaller the value, the more asymmetric and “elliptical/irregular” the cavity.

**Fig. 3:**
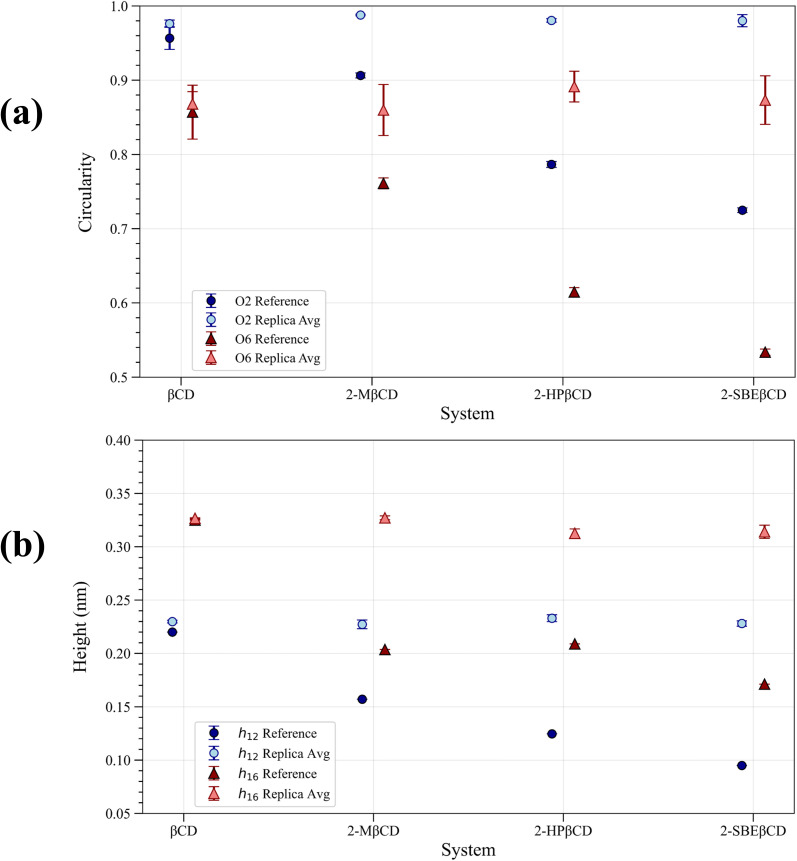
(a) Rim circularity of the cyclodextrin (CD) systems in the reference (water-only) and progesterone-bound states for the O_2_ (SHR) and O_6_ (PHR) rims (1 = perfect circle). Error bars represent reported uncertainties (reference values) and ±1 standard deviation across replicas. (b) Comparison of average height measurements (*h*_12_ and *h*_16_) for various cyclodextrin systems. Error bars represent reported uncertainties (reference values) and ±1 standard deviation across replicas.

In the reference βCD, both rims are relatively regular (SHR ≈ 0.96 and PHR ≈ 0.86), and it is observed that the O_2_ rim is inherently more circular than the O_6_, as can be seen in Fig. 3a. This structural asymmetry, *i.e.*, the higher rigidity of SHR and the greater flexibility of PHR, is maintained throughout the CD series. Upon addition of PROG, the circularity of both rims increases, although the extent of this change strongly depends on the type of derivative.

In βCD, the increase is minor (approximately 2–3%), whereas in substituted derivatives the improvement is substantial. For example, in 2-MβCD, SHR rises from ∼0.91 to ∼0.99 (+9%) and PHR from 0.76 to 0.88 (+15%). In 2-HPβCD this effect is stronger, with the SHR increasing from 0.79 to 0.98 (+25%) and the PHR from 0.61 to 0.90 (+46%). The greatest geometric rearrangement occurs in 2-SBEβCD, with the SHR increasing from 0.72 to 0.99 (36%) and the PHR from 0.53 to 0.87 (64%). This remarkable convergence of values of about 0.98 (SHR) and 0.87–0.90 (PHR) suggests that the PROG molecule acts as an internal template, providing symmetry and uniform arrangement of the glucopyranose rings and substituents.

Thus, PROG effectively “rounds” the asymmetric edges of the derivatives and converges the entry/exit geometry in different systems, while still maintaining the inherent advantage of the O_2_ rim over the O_6_ rim.

#### 3.2.3 Height of the rims

In our definition, *h*_12_ is the distance from the O_2_ plane to the O_1_ plane (secondary aperture), and *h*_16_ is the distance from the O_6_ plane to the O_1_ plane (primary aperture), as shown in Fig. S1. A larger value indicates that the aperture is more prominent or extends further along the axis, whereas a smaller value indicates that the aperture lies closer to the midplane, reflecting a tendency of the torus to flatten.

In the reference βCD, *h*_16_ is about 0.32–0.33 nm and *h*_12_ is about 0.22–0.23 nm, as illustrated in Fig. 3b. Thus, the primary aperture is inherently more prominent than the secondary aperture, preserving the classical conical shape of βCD. In the PROG-free derivatives, both heights decrease, in some cases dramatically.

In 2-MβCD, *h*_12_ falls to about 0.16 nm and *h*_16_ to about 0.20–0.21 nm; in 2-HPβCD, *h*_12_ drops as low as ∼0.125 nm; and in 2-SBEβCD, both apertures show the most pronounced depression (*h*_12_ ≈ 0.095 nm and *h*_16_ ≈ 0.17 nm). These results suggest that, in the absence of the PROG, bulky substituents compress the torus along the axis and drive the structure towards a flatter geometry.

In the presence of PROG, both heights increase and, more importantly, converge to relatively uniform values across all derivatives. For *h*_12_, all systems approach ∼0.23 nm. For *h*_16_, all derivatives reach ∼0.31–0.32 nm. This axial convergence suggests that PROG acts as an “internal mold”: by filling the interstitial space and maintaining continuous contact with the walls, it pushes the ring and substituents out of their recessed state, restores the torus thickness, and reestablishes the natural conicity.

By opening the compact torus of the derivatives along the axis, PROG brings the heights of the two apertures to values close to βCD. As a result, the inlet/outlet geometry becomes more uniform across the series, while the intrinsic prominence of the O_6_ aperture over O_2_ is still maintained.

#### 3.2.4 Surface-to-volume and moment of inertia

Comparing the total moment of inertia (*I*_tot_) and the ratio of the solvent-accessible surface area (SASA)^51^ of each CD to its volume (V) with the previously analyzed shape descriptors yields a coherent structural picture. In the PROG-free reference series, *I*_tot_ increases monotonically with substitution, whereas *S*/*V* decreases relative to βCD, indicating axial compression and rim irregularity, as seen in Table S1.

PROG inclusion reverses these trends and drives convergence across derivatives. Replica-averaged *I*_tot_ shows relative increases of about +0.9% (βCD), +8.8% (2-MβCD), +11.3% (2-HPβCD), and +5.6% (2-SBEβCD) *versus* reference. *S*/*V* likewise increases by ∼+0.5%, +3.8%, +7.2%, and +7.0% in the same order and collapses to a narrow band across all derivatives. Overall, guest binding reorganizes cyclodextrins toward thicker, rounder, and more symmetric tori, increasing both *I*_tot_ and *S*/*V* and homogenizing entry/exit geometry, features expected to facilitate stable guest passage and residence.

### 3.3 Hydration structure analysis

The radial distribution functions (RDFs) in the absence of PROG (Fig. S9a) indicate that water forms a dense first hydration shell around βCD, with a sharp primary peak at ∼0.25–0.30 nm and a secondary peak at ∼0.95–1.05 nm. Methylation in 2-MeβCD attenuates this near-contact feature and reduces the number of cavity waters, likely due to fewer hydrogen-bonding sites and increased wall hydrophobicity. In 2-HPβCD and 2-SBEβCD, the near-contact peak is largely suppressed and the principal maximum appears at ∼1.0 nm. Bulky substituents seem to occlude the rims and displace water into the outer shell.

**Fig. 4:**
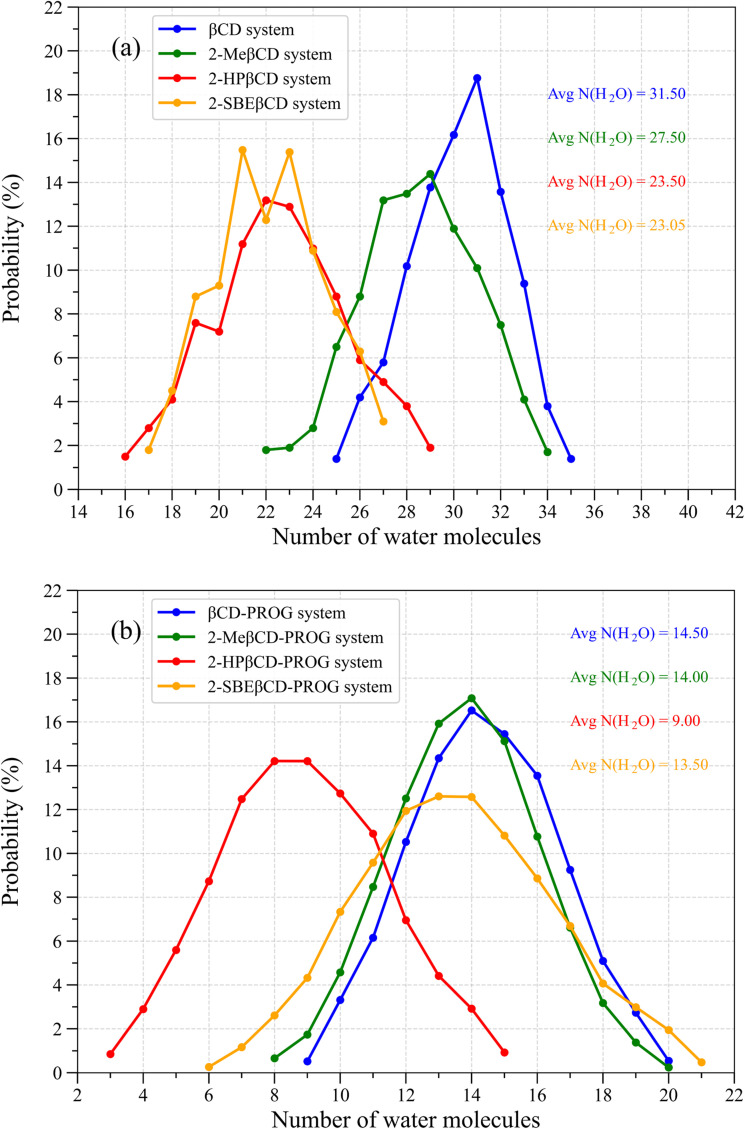
Probability distributions of the number of water molecules within 0.8 nm of the cyclodextrin center. (a) CD systems without PROG, (b) CD–PROG complexes.

A cutoff of *r* = 0.8 nm from the βCD center was adopted to define the inner hydration region. This value lies between the first minimum of the RDF (∼0.5 nm) and the second peak (∼0.95 nm) of the reference βCD–water system (Fig. S9a), approximately corresponding to the transition between the first and second hydration shells. It is also consistent with the experimentally established truncated-cone geometry of βCD, whose cavity has an approximate diameter of ∼0.60–0.66 nm and a height of ∼0.80 nm,^52–54^ thereby encompassing the full cavity interior together with the primary and secondary rim regions involved in guest hydration. The number of water molecules within *r* < 0.8 nm for βCD, 2-MeβCD, 2-HPβCD, and 2-SBEβCD is 31.5, 27.5, 23.5, and 23.05, respectively. The value for βCD is in good agreement with the other MD simulations.^55^ Because a mean alone does not capture distributional behavior, we also computed the probability distribution of water counts in this layer for all systems, as shown in Fig. 4. Loading of PROG reorganizes the RDFs (Fig. S9b), so that the contact peak vanishes and all systems exhibit a narrow maximum at 0.95–1.10 nm, indicating an outward shift of the hydration shell. Concomitantly, the population of waters with *r* < 0.8 nm is reduced by roughly half (to 14.5, 14.0, 9.0, and 13.5), with the strongest depletion in 2-HPβCD-PROG, consistent with deep guest insertion (Fig. 2) and rim occlusion.

By contrast, 2-SBEβCD-PROG retains some near-edge waters, as sulfonate groups can stabilize water at slightly larger distances. Thermodynamically, expelling confined waters can increase solvent entropy and removes unfavorable hydrophobic–water contacts, stabilizing the complex.^52,56–58^ The remaining waters interact primarily with the rims and polar substituents, and their hydrogen-bond network is more distorted than in bulk.

The tetrahedral order parameter for waters near the CDs (*r* < 0.8 nm) further resolves the quality of the hydrogen-bond network. As shown in Fig. S10a, without PROG all systems show distributions biased toward lower 〈*q*〉 (0.0–0.4). This is most pronounced in 2-HPβCD and 2-SBEβCD, where a probability maximum at *q* ≈ 0.1–0.2 indicates relatively disordered interfacial waters due to bulky/polar substituents. By contrast, βCD retains a subpopulation with higher local order, reflecting a simpler cavity and less-perturbed native H-bonding sites. Upon loading PROG, the probability at higher *q* values (≈0.4–1) decreases and density shifts to smaller *q* values, implying that waters remaining within *r* < 0.8 nm adopt more distorted, less-tetrahedral configurations. In effect, cavity occupation and rim blockage displace well-ordered water and leave water molecules bound to the rims and substituents, forming a frustrated network. The largest decrease in *q* is observed for 2-HPβCD-PROG (Fig. S10d), which has a minimal near-shell water count. In contrast, 2-SBEβCD-PROG retains edge waters, which, despite being relatively disordered, contribute to local electrostatic stabilization, as seen in Fig. S10e. Additional methodological details regarding the tetrahedral order parameter are provided in Section S4.

### 3.4 Pathway-dependent free-energy profiles and interaction analysis

#### 3.4.1 PMF

A thorough examination of the thermodynamic and kinetic aspects of guest encapsulation in and release from CD cavities is important for the rational design of CD-based drug-delivery systems. Thermodynamic parameters characterize the stability and binding affinity of drug–CD complexes, whereas kinetic parameters describe the rates of complex formation and dissociation. These processes depend on the molecular interactions and confined environment experienced by the guest within and near the CD cavity. Chemical modification of the CD rims can further modulate the steric and electronic characteristics of the binding environment, thereby altering complex stability, binding selectivity, solubility, and drug-release behavior.^59–61^ Such modifications may consequently facilitate the development of drug formulations with improved stability, bioavailability, or delivery performance.

The potential of mean force (PMF) represents the free energy profile of the system along the reaction coordinate *z*, which corresponds to the distance between the center of mass of PROG and the center of the CD cavity along the cavity axis. To compare the thermodynamic preference among different loading pathways, the PMF well depth of each pathway (Δ*G*_i_) was defined as the global minimum of the corresponding PMF profile relative to the unbound plateau. The relative thermodynamic preference between two pathways (*i*) and (*j*) was then assessed *via* their free energy difference ΔΔ*G*_*ij*_ = Δ*G*_*i*_ − Δ*G*_*j*_, where a more negative value indicates a deeper PMF well for pathway (i). Each PMF profile was referenced to zero using the mean value in the unbound plateau region at *z* ≥ 1.5 nm. The resulting values represent pathway-specific estimates of relative binding stability rather than equilibrium populations of the complete system, because spontaneous interconversion between distinct binding modes was not observed within the simulation timescale.

**Fig. 5:**
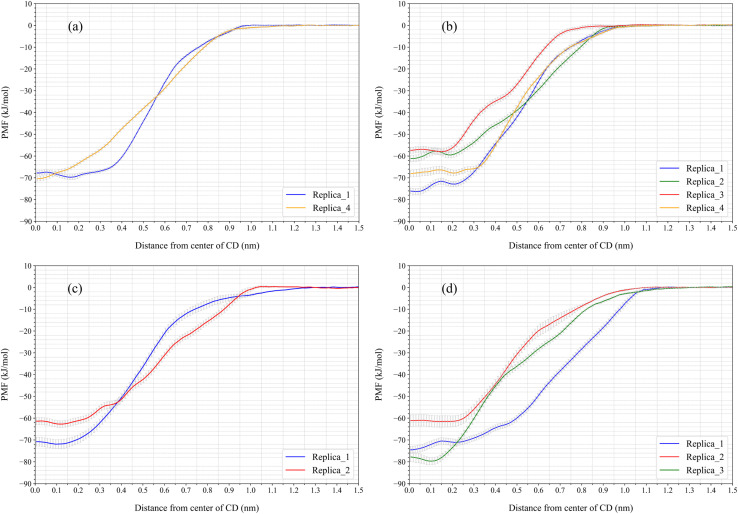
Representative PMF profiles corresponding to the distinct PROG loading mechanisms observed for (a) βCD, (b) 2-MeβCD, (c) 2-HPβCD, and (d) 2-SBEβCD. For mechanisms assigned to the same loading-pathway category across multiple replicas, one representative PMF profile is shown for clarity.

For βCD (Fig. 5a), the SHR pathway represented by Replicas 1–3 exhibited a PMF well depth of −69.77 kJ mol^−1^ relative to the unbound plateau. A broad low-free-energy region was observed between approximately 0 and 0.3 nm, indicating a relatively broad bound basin along the selected coordinate. The PHR pathway represented by Replicas 4 and 5 exhibited a closely comparable PMF well depth of −70.65 kJ mol^−1^. The difference between the two PMF minima is only 0.88 kJ mol^−1^ (≈0.35 *k*_B_*T* at 300 K), which is comparable to the expected statistical uncertainty of the PMF reconstruction. The two βCD pathways should therefore be regarded as indistinguishable within the resolution of the present calculations, and the small difference should not be interpreted as evidence of a thermodynamic preference for either pathway. Instead, the two βCD loading modes should be considered thermodynamically comparable within uncertainty.

The main distinction between the two pathways lies in the shape of the PMF profiles and the associated binding geometry. The absence of a broad low-free-energy region in the PHR pathway suggests that PROG tends to penetrate deeper into the cavity, consistent with the distance analysis between PROG and the cavity center (Fig. 2). In addition, Replica 4 shows a more gradual ascent from the energy minimum along the dissociation coordinate compared with Replica 1, which exhibits a steeper and sharper free-energy increase.

In the case of 2-MeβCD, as illustrated in Fig. 5b, the SHR pathways involving the O20-side (Replica 1) and O3-side (Replica 4) exhibited PMF well depths of −75.34 and −68.14 kJ mol^−1^, respectively. These two mechanisms also exhibited nearly identical energy slopes along the penetration pathway. In contrast, binding from the PHR, through either the O20-side (Replica 3) or the O3-side (Replica 2), resulted in shallower PMF minima (−57.52 and −60.90 kJ mol^−1^, respectively) and broadly similar profile shapes along the selected coordinate. Overall, the SHR-associated pathways exhibited steeper free-energy profiles than the PHR-associated pathways.

Among the sampled 2-MeβCD pathways, Replica 1 exhibited the deepest PMF minimum, with Δ*G* = −75.34 kJ mol^−1^, being preferred over the PHR pathways with ΔΔ*G* = −17.82 kJ mol^−1^ relative to Replica 3 and ΔΔ*G* = −14.44 kJ mol^−1^ relative to Replica 2. Replica 1 is also preferred over the SHR/O3 pathway, with ΔΔ*G* = −7.20 kJ mol^−1^ relative to Replica 4.

For 2-HPβCD (Fig. 5c), the PMF profiles show significant differences between the two loading pathways. The SHR/O20 pathway represented by Replica 1 exhibited a PMF well depth of −71.45 kJ mol^−1^, whereas the PHR/O3 pathway represented by Replica 2 exhibited a shallower PMF minimum of −62.88 kJ mol^−1^. Both mechanisms exhibit a relatively flat PMF region between 0–0.14 nm from the center of the 2-HPβCD, corresponding to the preferred binding region of PROG in this carrier. The deeper PMF well observed for Replica 1 indicates a lower free-energy minimum along the sampled SHR/O20 pathway under the present reaction-coordinate definition. It seems that this mechanism is potentially facilitated by the formation of additional hydrophobic and hydrogen-bonding interactions upon inclusion through the wider opening. Replica 1 represents the thermodynamically most favorable binding mode, with ΔΔ*G* = 8.57 kJ mol^−1^ (∼3.5 *k*_B_*T* at 300 K) relative to Replica 2, clearly highlighting the thermodynamic preference for the SHR/O20 pathway.

In the case of 2-SBEβCD, loading of PROG through the O3-side, either *via* the SHR (Replica 3) or PHR (Replica 1) rims, exhibited the highest thermodynamic favorability. Among these two pathways, the SHR/O3 rim demonstrated a superior energetic preference, as illustrated in Fig. 5d. The PHR/O3 pathway exhibited a less steep free-energy increase along the dissociation coordinate, whereas the SHR/O3 pathway showed the deepest PMF minimum among all monitored profiles.

In addition, loading profiles of SHR mechanisms (*i.e.*, Replica 2 and 3) have nearly identical patterns of slopes. Replica 3 with Δ*G* = −80.10 kJ mol^−1^ represents the strongly preferred binding mode, with ΔΔ*G* = −5.52 kJ mol^−1^ relative to Replica 1 Δ*G* = −74.58 kJ mol^−1^ and ΔΔ*G* = −18.30 kJ mol^−1^ (∼7.3 *k*_B_*T* at 300 K) relative to Replica 2 Δ*G* = −61.80 kJ mol^−1^, indicating that Replica 2 exhibits a shallower PMF minimum and is therefore energetically less favorable among the sampled pathways.

As shown in Fig. S11, a key finding of thermodynamic analysis is that short-range non-bonded vdW interactions are much more dominant in PROG binding to all carriers than short-range non-bonded coulombic interactions. Notably, although functionalization of βCD occurs solely at position 2 and at the SHR, a significant increase in vdW interaction energy at short distances (less than 0.4 nm) is observed across all PROG entry mechanisms, whether through the SHR or PHR rim. In fact, the magnitude of the vdW interaction energies in βCD range from 110 to 130 kJ mol^−1^ at distances below 0.4 nm across all entry mechanisms, whereas in the case of 2-HPβCD and 2-SBEβCD these values range from 130 to 150 kJ mol^−1^. This finding suggests that structural modifications introduced by bulky and polarizable groups, such as hydroxypropyl (in 2-HPβCD) and sulfobutylether (in 2-SBEβCD), induce non-local alterations in the overall spatial configuration of the host cavity. These changes increase the effective contact surface between PROG and the inner CD wall along the entire insertion pathway. As a result, enhanced vdW interactions are observed for both SHR and PHR entry mechanisms. Consequently, R_2_ functionalization may thermodynamically stabilize the host–guest complex through improved spatial complementarity and strengthened non-covalent contacts.

#### 3.4.2 Short-range non-bonded interaction energies

**Fig. 6:**
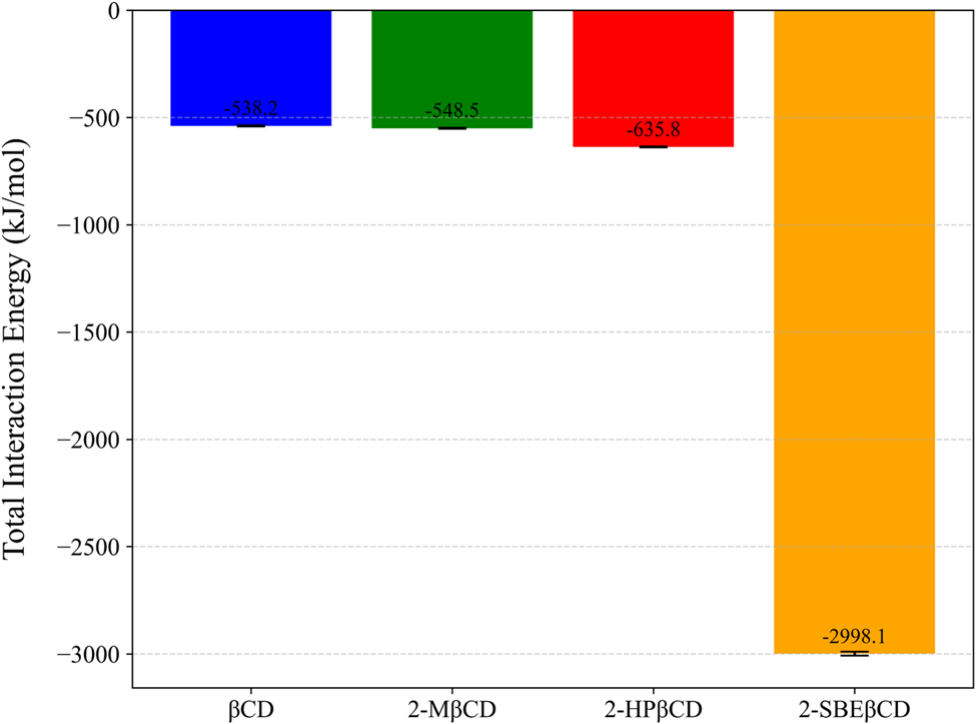
Total short-range non-bonded interaction energies (short-range vdW + short-range coulombic) between the PROG–CD complexes and surrounding water molecules across the different simulated systems. Negative values indicate net attractive short-range non-bonded interactions between the complexes and water. Data represent the mean values over five independently simulated initial configurations for each carrier, and error bars indicate the standard error of the mean (SEM).

Examination of the short-range interaction energies between the PROG–CD complexes and water molecules in the environment shows that the nature of the chemical substitution on the CD ring significantly influences the complex–water interaction pattern, as shown in Fig. 6. The reported interaction energies comprise short-range vdW and coulombic components, whose sum provides a direct measure of the short-range non-bonded interactions between each PROG–CD complex and its surrounding water molecules, as summarized in Table S2.

Among the four carriers analyzed, βCD (being unsubstituted and moderately polar because of its surface hydroxyl groups, while devoid of significantly polar or ionic substitutions) demonstrates the least negative mean total interaction energy (around −538.2 kJ mol^−1^), with vdW interactions constituting just over 51.5% (−276.66 kJ mol^−1^), and coulombic contributions making up the remaining 48.5% (−261.55 kJ mol^−1^). This balance indicates its moderate ability to participate in hydrogen bonding through hydroxyl groups, whereas the absence of charged or highly polar substituents restricts the degree of electrostatic stabilization in the aqueous surroundings.

2-MβCD displays a slightly stronger interaction (−548.5 kJ mol^−1^), primarily due to increased nonpolar interactions. Methyl groups increase the nonpolar character of the CD, thereby strengthening vdW interactions with nearby water molecules (−313.48 kJ mol^−1^, which is more than 57% of the total interaction energy). However, the lack of hydrogen bonding capability (due to the loss of the OH group at the O_2_ position) limits additional stabilization, as no significant gain in coulombic (−234.98 kJ mol^−1^, which is less than 43%) contribution compared to βCD is observed.

In contrast, 2-HPβCD exhibits a more negative interaction energy (−635.5 kJ mol^−1^), reflecting improved stabilization. This enhanced stabilization arises from the presence of hydrophilic hydroxypropyl groups that can form multiple hydrogen bonds with water. This allows for the retention of significant vdW (−364.42 kJ mol^−1^, which is more than 57%) interactions while increasing the absolute amount of the coulombic interactions compare to βCD (−271.4 kJ mol^−1^, which is less than 43%). This interaction pattern reflects contributions from both vdW contacts and short-range coulombic interactions, the latter being consistent with interactions between the hydroxyl groups of the hydroxypropyl substituents and surrounding water molecules.

The most negative mean total short-range complex–water interaction energy was observed for 2-SBEβCD (−2998.1 kJ mol^−1^). This large negative value was predominantly associated with the short-range coulombic component, which accounted for more than 95% of the total interaction energy (−2867.15 kJ mol^−1^). To clarify the origin of this strong stabilization, the interaction energies were further decomposed into pairwise group–group contributions. This analysis revealed that the dominant contribution arises from the interaction between the sulfonate groups and surrounding water molecules. In particular, the coulombic SO_3_^−^–water interaction remained consistently around −2206 kJ mol^−1^ across the five independent replicas, whereas the corresponding Lennard-Jones term was much smaller and opposite in sign (approximately +231 kJ mol^−1^). By contrast, the interaction between the sulfonate groups and the remaining non-solvent components of the system was considerably weaker, with average coulombic and Lennard-Jones contributions of only about −133 and −63 kJ mol^−1^, respectively. These results demonstrate that the exceptionally large interaction energy of 2-SBEβCD reflects strong and reproducible electrostatic hydration of the sulfobutylether substituents rather than direct host–guest interactions. This interpretation is consistent with the previous structural analyses, which showed that 2-SBEβCD possesses the most polar and strongly hydrated interfacial environment among the carriers examined.

#### 3.4.3 Hydrogen bond

Hydrogen bonds between water molecules and CDs play a key role in determining the dynamic behaviour and thermodynamic stability of drug complexes.^62,63^ To better understand these interactions, we investigated the distribution and average number of hydrogen bonds in two radial regions around the geometric center of the CD: the inner cavity region (0–0.8 nm) and the peripheral region (0.8–1.1 nm). These boundaries were selected based on the positions of the first and second minima observed in the RDF of water around the CD cavity, as shown in Fig. S9. These regions represent the direct interaction of water with the inner surface and outer edges of the CD, respectively.

**Fig. 7:**
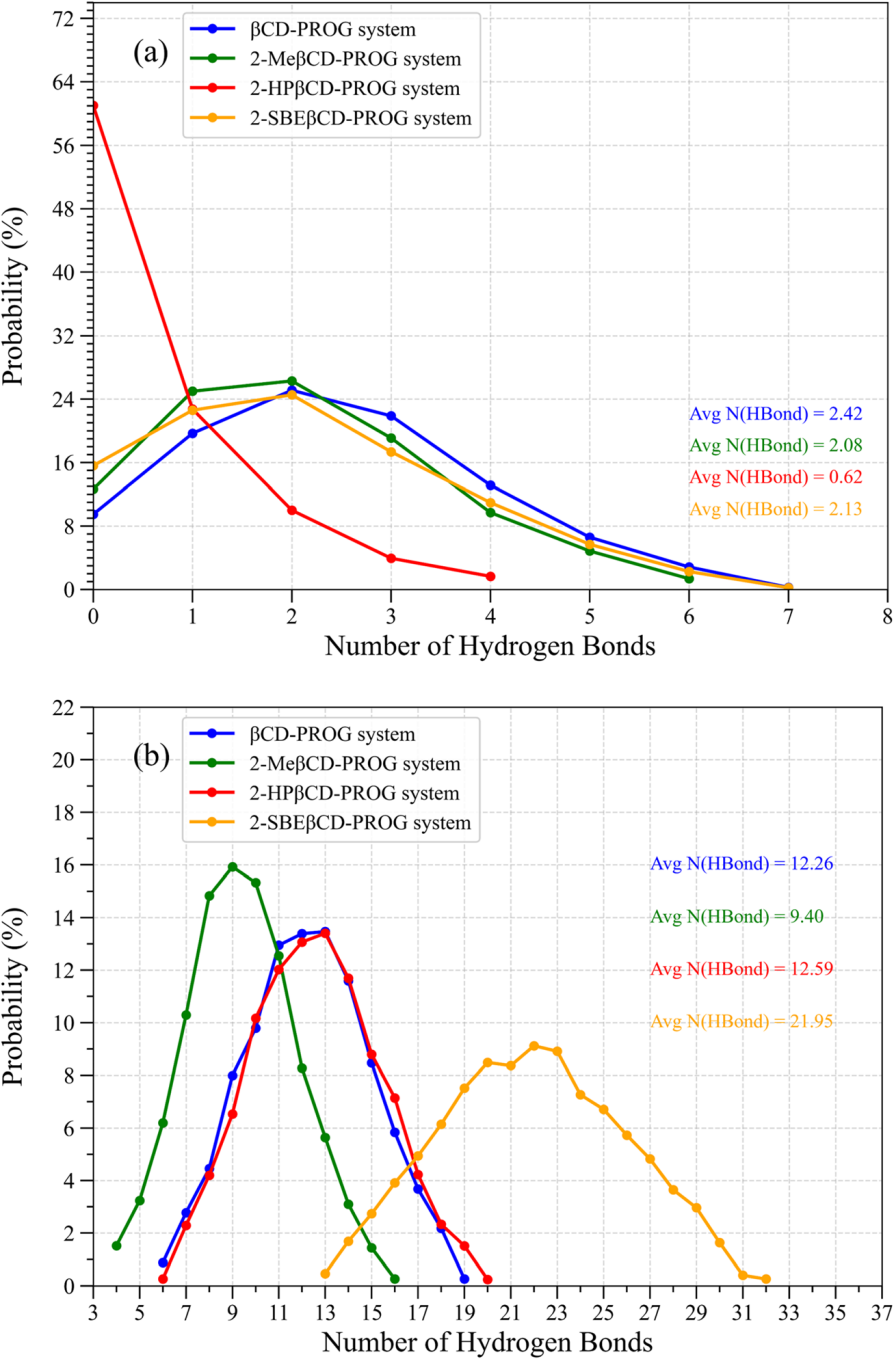
Distribution and average number of hydrogen bonds between water molecules and CDs in different CD–PROG complexes within (a) 0–0.8 nm and (b) 0.8–1.1 nm from the CD cavity center.

Fig. 7a shows that in the inner region, the βCD-PROG, 2-SBEβCD-PROG, and 2-MeβCD-PROG systems formed an average of 2.42, 2.13, and 2.08 hydrogen bonds, respectively. In contrast, 2-HPβCD-PROG showed the weakest interaction, forming an average of only 0.62 bonds. This significant decrease in 2-HPβCD is due to the presence of hydroxypropyl-substituted chains at the R_2_ position. Although these chains have polar –OH groups, their amphiphilic nature, and especially their spatial structure, result in steric hindrance at the cavity entrance, limiting the accessibility of water molecules to the central region without fully blocking the entry. In other words, these chains act more as a steric barrier than an effective source of hydrogen bonding in the center of the cavity.

Significant differences were observed in the peripheral region (0.8–1.1 nm), as seen in Fig. 7b. The 2-SBEβCD-PROG system had the highest number of hydrogen bonds, with an average of 21.95 bonds, and was clearly distinguished from the other systems. This can be attributed to the presence of the anionic sulfobutylether substituent groups, which create a highly charged environment and facilitate the formation of an extensive hydrogen-bond network with the surrounding water molecules. In contrast, the 2-MeβCD-PROG system recorded the lowest number of bonds in this region (an average of 9.40) due to the lack of polar groups at the R_2_ position, which increases hydrophobicity. The βCD-PROG and 2-HPβCD-PROG systems showed averages of 12.26 and 12.59 bonds respectively, reflecting the balance between OH group presence (in βCD) and the dual effects of the substituent chain in 2-HPβCD.

Comparing the two radial regions shows that, while the chemical changes to the CD affect the number of hydrogen bonds in both regions, the main effect of the substituent groups occurs in the peripheral region. Notably, the charged groups in 2-SBEβCD create an electrostatic environment that retains water molecules and forms stable bonding networks, even at peripheral distances (*i.e.*, between 0.8 and 1.1 nm from the center).

From a thermodynamic point of view, the reduction in hydrogen bonds in the inner region after PROG loading results in the release of bound water and an increase in entropy. This contributes to the greater stability of the complex. In contrast, the remaining water molecules in the rim region, especially in the presence of charged or polar groups, may influence the local environment associated with drug release and contribute to structural stabilization.

## 4 Conclusion

In this work, molecular dynamics simulations combined with PMF-based free energy calculations were employed to investigate the encapsulation of PROG within native βCD and three non-toxic derivatives (2-MeβCD, 2-HPβCD, and 2-SBEβCD) with particular emphasis on the influence of R_2_ functionalization on inclusion mechanisms, structural stability, and solvent-mediated interactions.

PMF calculations revealed pathway-dependent free-energy-well depths ranging from −57.52 to −80.10 kJ mol^−1^. Comparison of ΔΔ*G* values between competing pathways within each carrier demonstrated that specific loading mechanisms are strongly thermodynamically preferred, with particularly pronounced directional selectivity observed for 2-HPβCD (SHR/O20 pathway, −71.45 kJ mol^−1^) and 2-SBEβCD (SHR/O3 pathway, −80.10 kJ mol^−1^). Geometric and structural descriptors derived from the gyration tensor, rim circularity, and aperture heights consistently demonstrated that PROG inclusion drives host structural reorganization toward thicker, more symmetric toroidal conformations, an effect most pronounced in derivatives bearing bulky and flexible substituents. Complementary analyses of solvent-accessible surface area, moment of inertia, and radial distribution functions confirmed that PROG loading is accompanied by partial cavity dehydration and displacement of confined water molecules, contributing to thermodynamic stabilization through solvent entropy gain.

Hydrogen bond analysis showed that chemical substitutions primarily modulate water structuring in the peripheral region of the CD, while interaction energy decomposition established that vdW interaction dominate the direct short-range PROG–CD interaction across all carriers. The markedly more negative total short-range complex–water interaction energy observed for 2-SBEβCD (−2998.1 kJ mol^−1^) originated predominantly from electrostatic interactions between its charged sulfonate groups and surrounding water rather than from enhanced direct PROG–CD interactions. This distinction highlights the importance of substituent hydration when evaluating the aqueous behavior of functionalized cyclodextrin carriers.

These findings provide molecular-level insights relevant to the rational design of CD-based delivery systems and illustrate how substituent-induced geometric and energetic effects at the CD rim influence encapsulation behavior and solvent interactions. It should be noted that the binding pathways and thermodynamic preferences reported here reflect the specific assumptions, initial configurations, the force field employed, and simulation protocol adopted in this study. Alternative loading mechanisms may emerge under different initial placement schemes or with other force fields, and their systematic exploration would constitute a valuable extension of the present work.

## Author contributions

Faezeh Mobini: methodology, investigation, formal analysis, data curation, visualization. Marina Provenzano: conceptualization, writing – original draft, formal analysis. Nada Alghamdi: conceptualization, writing – original draft, formal analysis. Matteo Fasano: methodology, conceptualization, writing – review and editing, formal analysis. Mokhtar Ganjali Koli: conceptualization, methodology, validation, supervision, project administration, writing – original draft, writing–review and editing.

## Conflicts of interest

The authors declare no competing financial interest.

## Supplementary Material

RA-OLF-D6RA04566A-s001

RA-OLF-D6RA04566A-s002

## Data Availability

All of the initial inputs and the data supporting this article have been included as part of the supplementary information (SI). Supplementary information: additional structural and interaction-energy analyses of the host–guest systems, including representative loading snapshots of progesterone within β-cyclodextrin derivatives, time evolution of host–guest distances, structural descriptors of cyclodextrin geometry, solvent-accessible surface area/volume and moment-of-inertia analyses, radial distribution functions and tetrahedral order parameters describing hydration structure, and non-bonded interaction energy decompositions between progesterone, cyclodextrins, and water molecules (PDF). See DOI: https://doi.org/10.1039/d6ra04566a.
