# Supplementary material for: Host–guest complexation of progesterone with β-cyclodextrin derivatives: hydration structure, binding thermodynamics, and loading pathways from molecular dynamics simulations
Source: RSC Adv. 2026 Jul 30;16(39):43989–4002. doi: 10.1039/d6ra04566a (PMC13420253; doi:10.1039/d6ra04566a)
Supplement: Supplementary file 1 [file RA-016-D6RA04566A-s002.pdf]

Supplementary Information (SI) for RSC Advances.
This journal is © The Royal Society of Chemistry 2026

# Supplementary information: Host–guest complexation of progesterone with β-cyclodextrin derivatives: hydration structure, binding thermodynamics, and loading pathways from molecular dynamics simulations

**Faezeh Mobini[a], Marina Provenzano[b], Nada Alghamdi[b], Matteo Fasano[b], Mokhtar Ganjali Koli [c,d,*]**

[a]Venom and Biotherapeutics Molecules Lab, Medical Biotechnology Department, Biotechnology Research Center, Pasteur Institute of Iran, Tehran, Iran

[b]Department of Energy, Politecnico di Torino, Corso Duca degli Abruzzi 24, 10129 Torino, Italy

[c]Department of Chemistry, University of Kurdistan, Sanandaj, Iran

[d]Computational Chemistry Laboratory, Kask Afrand Exire Ltd., Sanandaj, Iran

***Corresponding Author:**

Email: m.ganjalikoli1360@gmail.com

*Contents*

**Figure S1:** Nomenclature and definitions used in this article for structural parameters of CDs……….……..…………………………………………………………….………….**4**

**Figure S2:** Five distinct initial placements of PROG for each CD molecule, each leading to the construction of an independent simulation system……………………………….…………**5**

**Section S1. Additional Simulation Details**……………………………….………..**6**

**Figure S3:** Time evaluation the distance between Progesterone and the center of CD molecules……………………………………………………………….……….….**8**

**Figure S4:** Representative snapshots of the moment of entry and loading mechanism of PROG into the βCD cavity (left), along with their final configurations (right) in different replicas………………………………………………………………..………..…..**10**

**Figure S5:** Representative snapshots of the moment of entry and loading mechanism of PROG into the 2-MβCD cavity (left), along with their final configurations (right) in different replicas………………………………………………………………….………………**12**

**Figure S6:** Representative snapshots of the moment of entry and loading mechanism of PROG into the 2-HPβCD cavity (left), along with their final configurations (right) in different replicas………………………………………………………………….……………**14**

**Figure S7:** Representative snapshots of the moment of entry and loading mechanism of PROG into the 2-SBEβCD cavity (left), along with their final configurations (right) in different replicas…………………………………………………………..………………**16**

**Section S3. Additional Structural Characteristics of CDs**…………………….………………**17**

**Figure S8:** Global shape descriptors derived from the gyration tensor (b, c, $\kappa^2$) for the cyclodextrin systems in water (reference) and in progesterone-bound simulations (replica average). Error bars represent reported uncertainties (reference values) and ±1 standard deviation across replicas…**18**

**Table S1:** The surface-to-volume ratio (S/V) and moment of inertia ($I_{tot}$) of carriers in different simulated systems…………………………………………………………..…….…….**20**

**Figure S9:** Radial distribution function (RDF) of water around CDs in (a) CDs without PROG, and (b) in PROG containing systems………..…………………………………………..**21**

**Section S4. Tetrahedral Order Parameter: Definition and Interpretation**…………………**22**

**Figure S10:** Tetrahedral order parameter (q) of water molecules within r < 0.8 nm from the cavity center of CDs (a) CDs without PROG, (b) different replicas of the βCD–PROG system, (c)

different replicas of the 2-MeβCD–PROG system, (d) different replicas of the 2-HPβCD–PROG system, and (e) different replicas of the 2-SBEβCD–PROG system. ……………………………**23**

**Figure S11:** Short-range non-bonded (a) van der Waals (vdW) and (b) Coulombic interaction energies between PROG and the CDs as a function of the distance from the CD cavity center.……………………………………………..……………………………………...**24**

**Table S2:** Decomposition of non-bonded interaction energies (van der Waals and Coulomb) between progesterone–cyclodextrin (PROG–CD) complexes and water molecules. Values are reported separately for each replica across the different cyclodextrin systems………..……….**25**

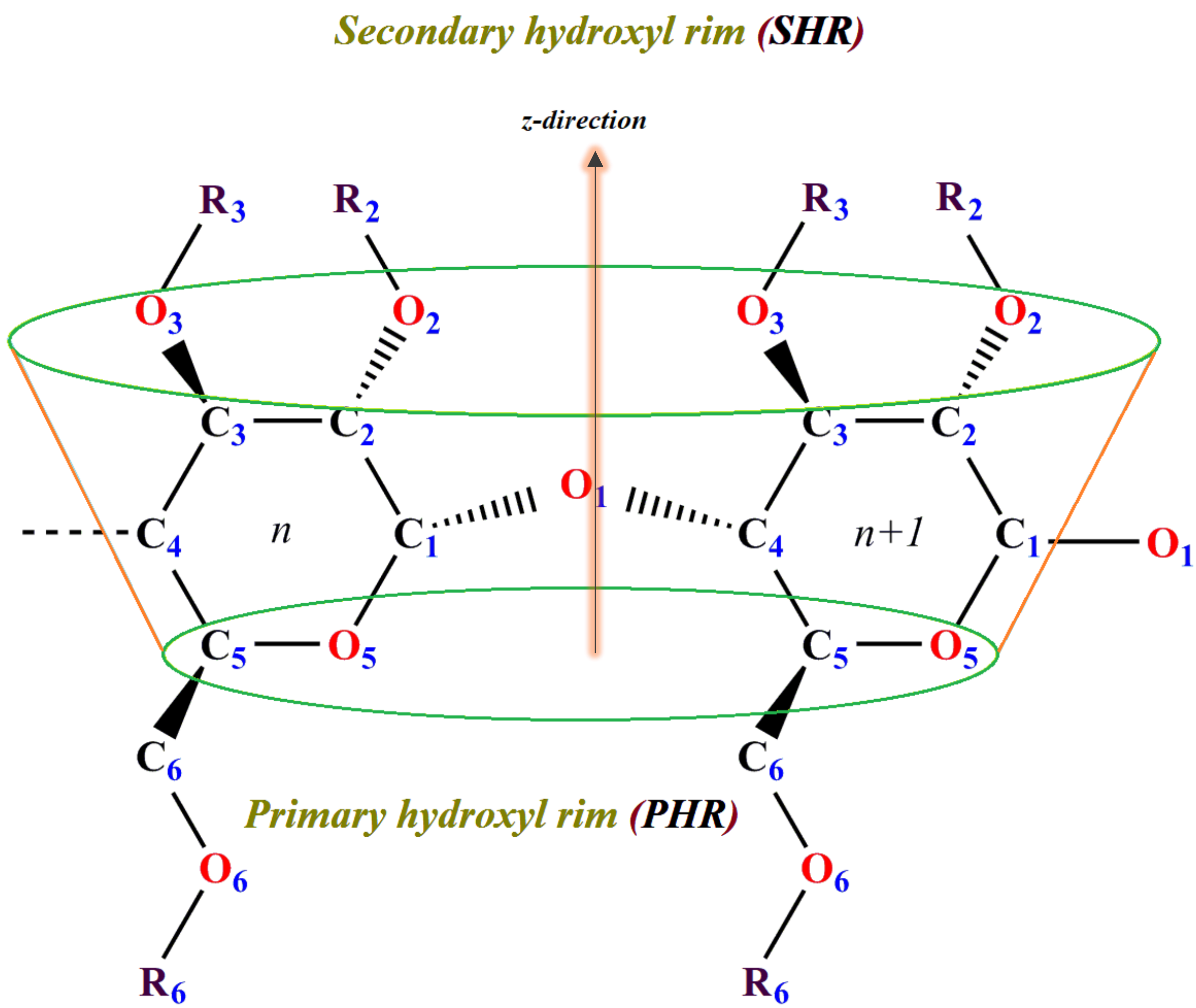


**Figure S1**: Nomenclature and definitions used in this article for structural parameters of CDs.

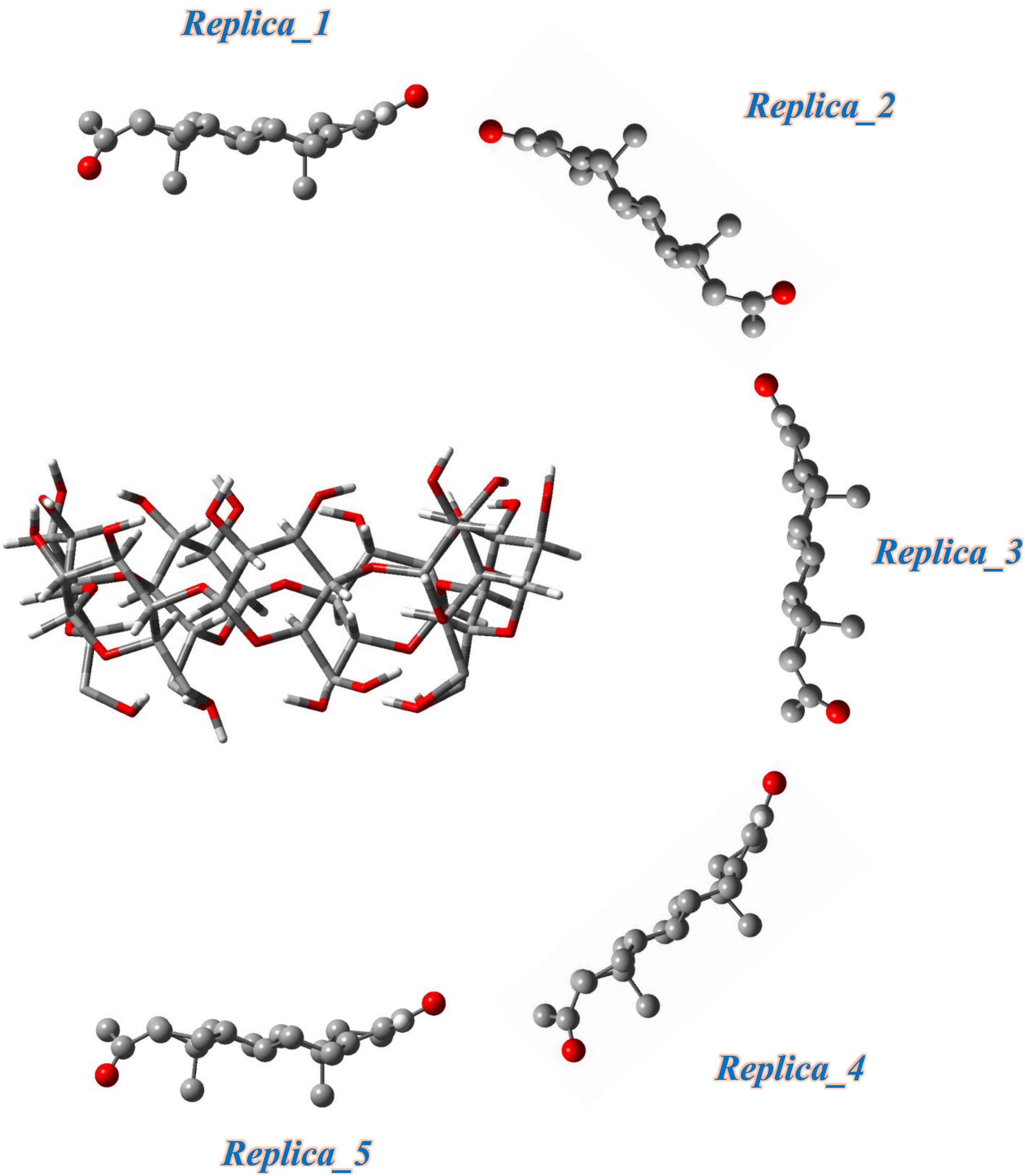


**Figure S2:** Five distinct initial placements of PROG for each CD molecule, each leading to the construction of an independent simulation system.

## Section S1. Additional Simulation Details

This section provides additional methodological details supporting the molecular dynamics simulations described in the main text.

### S1.1 Initial Configurations of PROG Relative to CDs

To ensure adequate sampling of possible host–guest approach pathways, five predefined initial orientations of the PROG molecule were generated relative to the CD cavity (**Figure S2**). These configurations were designed to represent distinct spatial arrangements of PROG around the host molecule prior to complex formation. This number was guided by the symmetry of the host–guest system: progesterone presents two chemically distinct termini (the O3 side and the O20 side), while each CD carrier offers two distinct entry rims (SHR and PHR) capable of accommodating the guest, yielding a maximum of four topologically distinct loading mechanisms, consistent with the pathway classification used in Section 3.4. The five predefined orientations therefore exceed this combinatorial maximum, providing redundant coverage to ensure that all mechanistically accessible pathways could be sampled, while each configuration was designed to differ maximally from the others so as to scan the relevant configurational space as broadly as possible.

### S1.2 Definition of Reference Atoms for Restraints and Reaction Coordinate

The $\mathbf{O_1}$ atoms of the CD molecules were selected as reference points for applying positional restraints and defining the reaction coordinate during the umbrella sampling simulations. These atoms are located at the middle rim of the CD structure and provide a consistent geometric reference for tracking the position of PROG along the cavity axis. A schematic representation of the selected atoms is provided in **Figure S1**.

### S1.3 Extended Simulation Time in Selected Replicas

In most cases, a production simulation time of 200 ns was sufficient to achieve stable inclusion of PROG within the CD cavity. However, in two specific instances, extended simulation times were required. In Replica 3 of the 2-HPβCD system and Replica 4 of the 2-SBEβCD system, the simulations were extended to 500 ns, as complete loading occurred at approximately 466 ns and 434 ns, respectively. This behavior indicates that these initial configurations required a longer exploration of conformational space before reaching a stable bound state. Despite the longer equilibration time, the overall loading mechanisms observed in these replicas remained consistent with those identified in other systems.

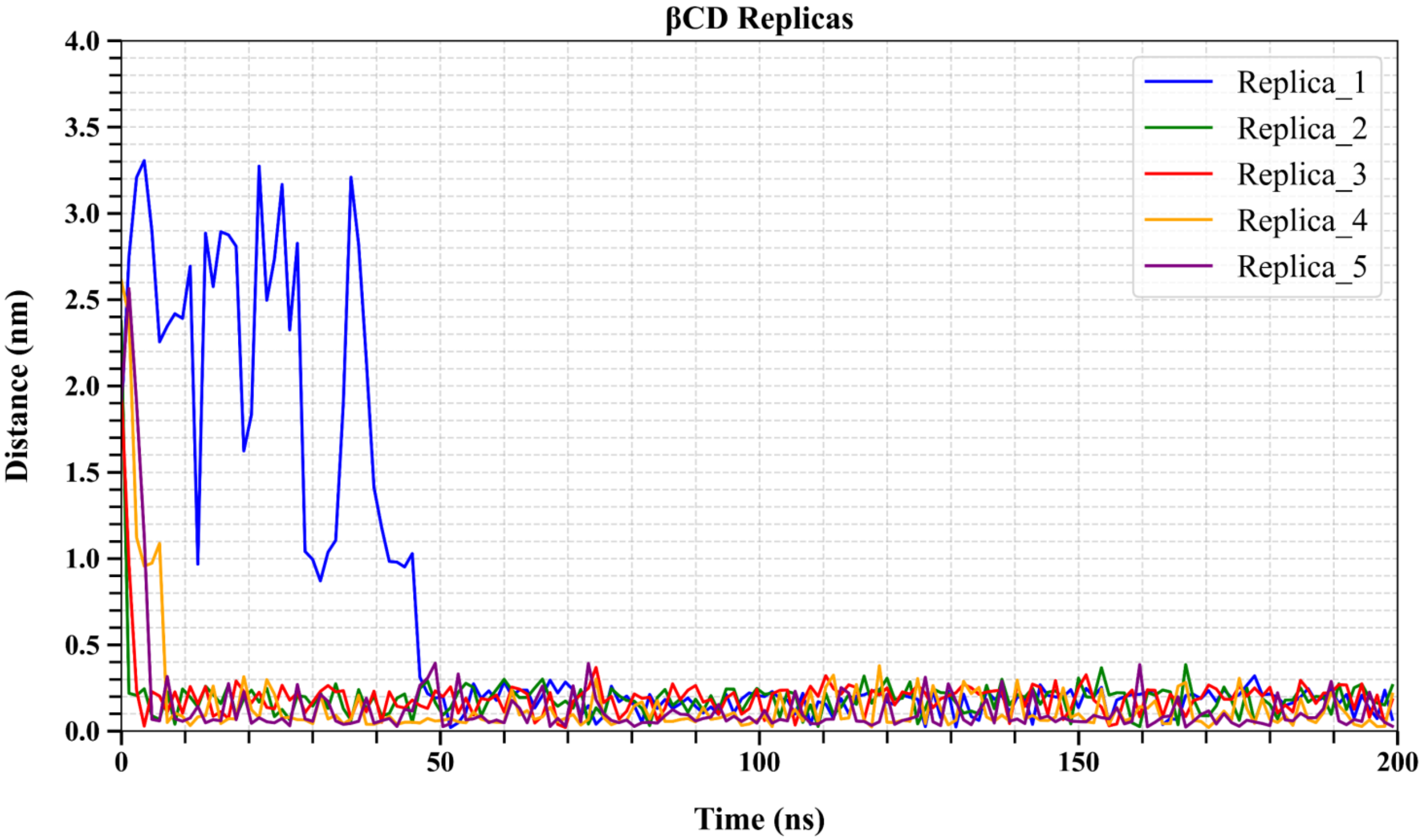
βCD Replicas
Replica_1
Replica_2
Replica_3
Replica_4
Replica_5
Distance (nm)
Time (ns)
0.0
0.5
1.0
1.5
2.0
2.5
3.0
3.5
4.0
0
50
100
150
200

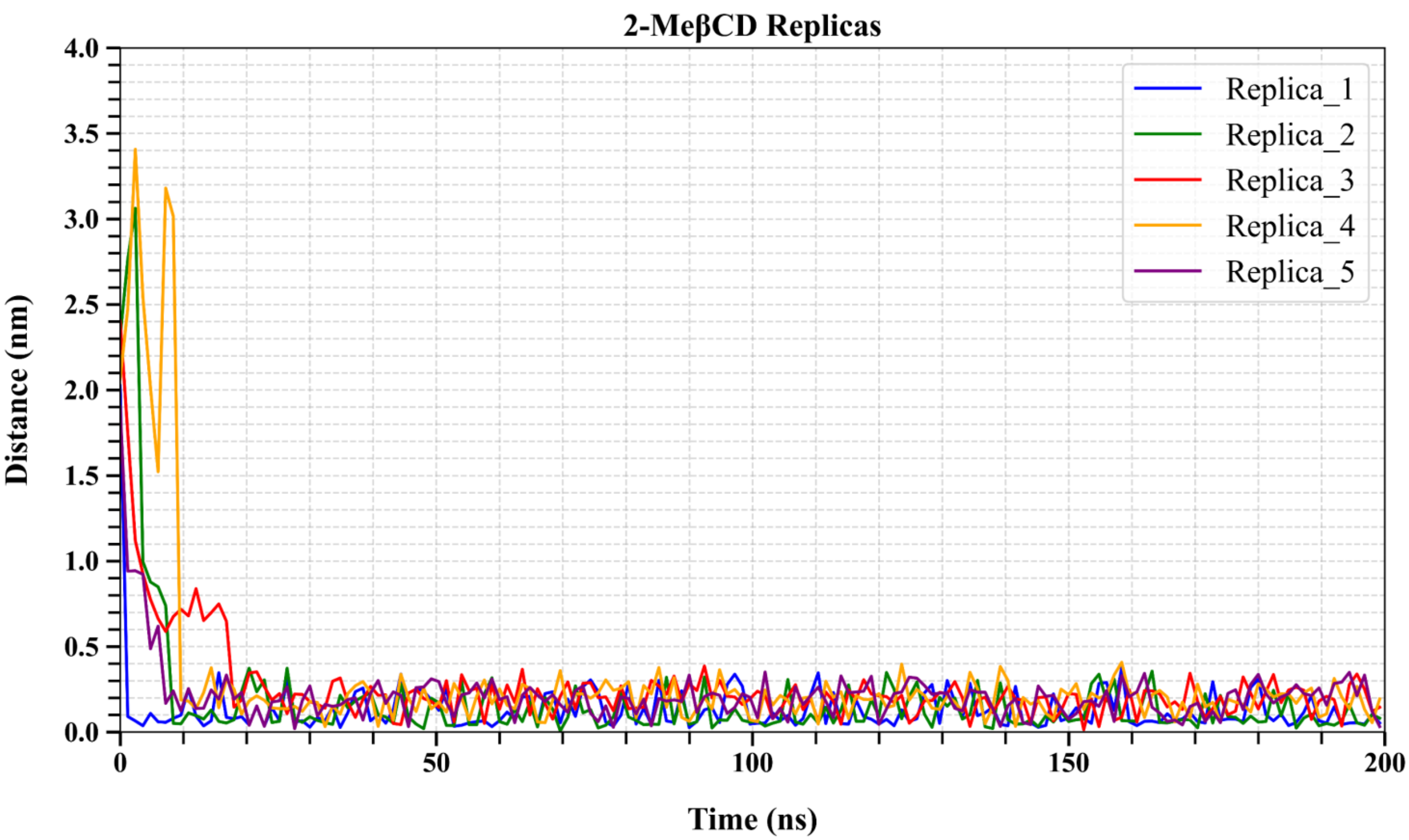
2-MeβCD Replicas
Replica_1
Replica_2
Replica_3
Replica_4
Replica_5
Distance (nm)
Time (ns)
0.0
0.5
1.0
1.5
2.0
2.5
3.0
3.5
4.0
0
50
100
150
200

***Continued***

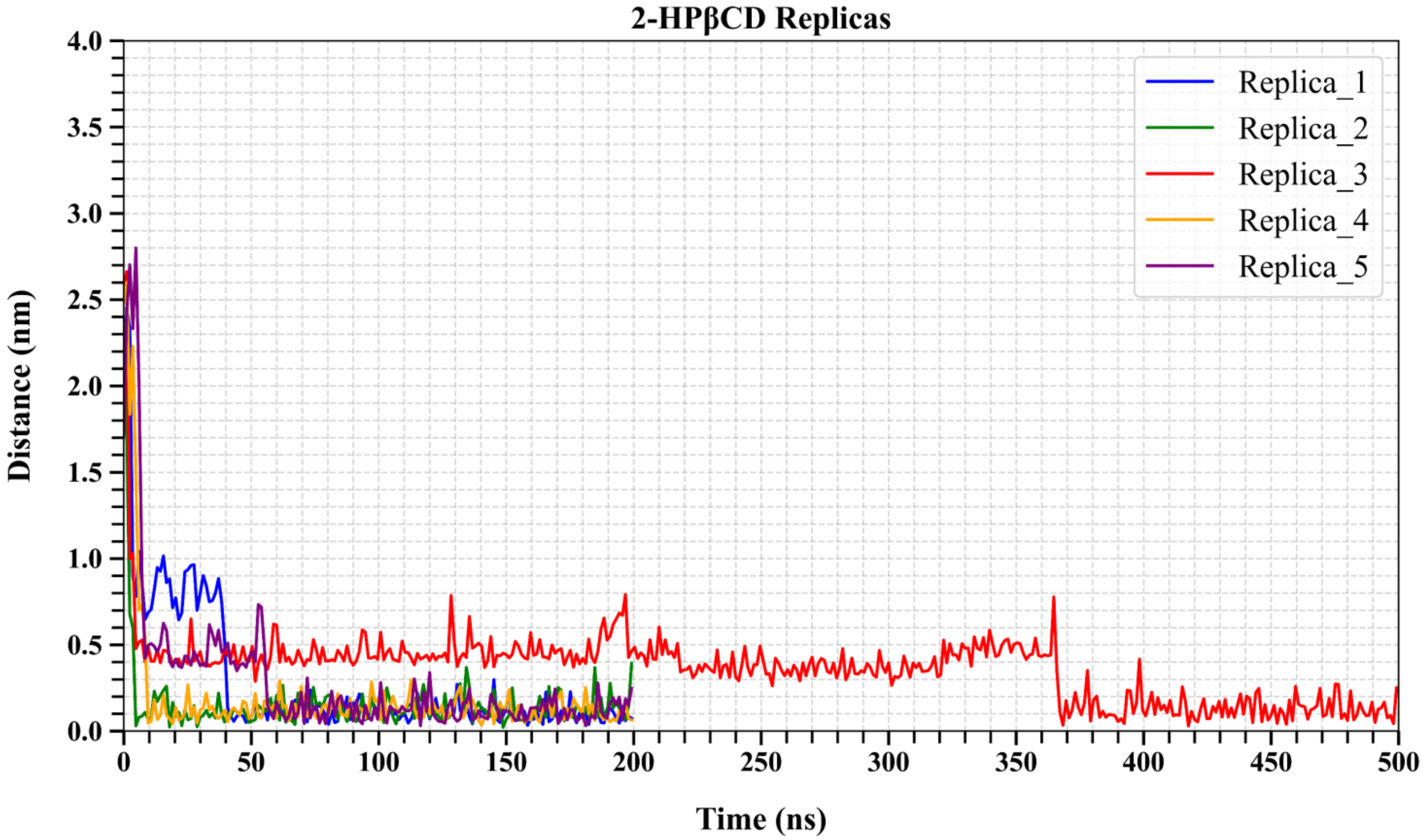


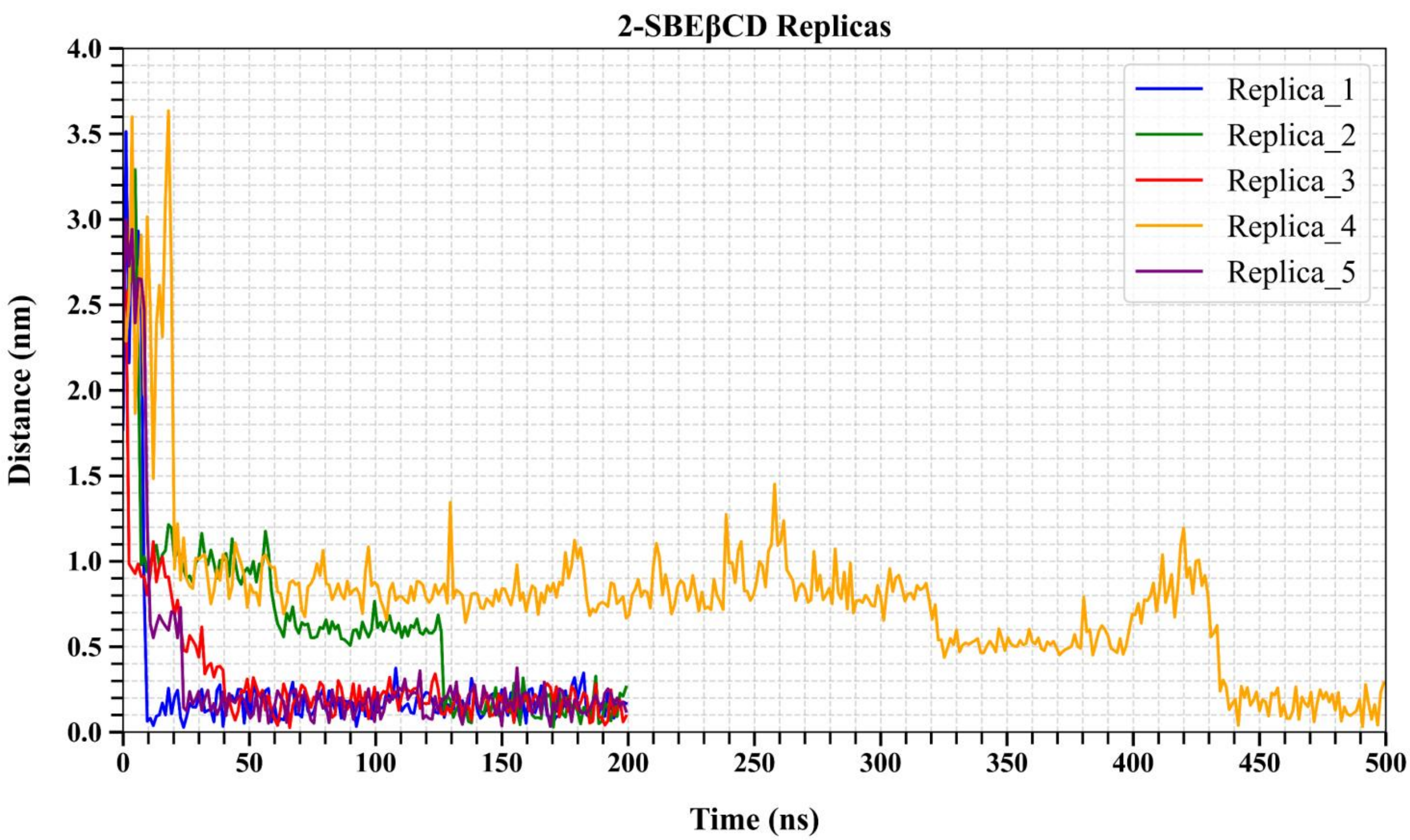


**Figure S3.** Time evaluation the distance between Progesterone and the center of CD molecules.

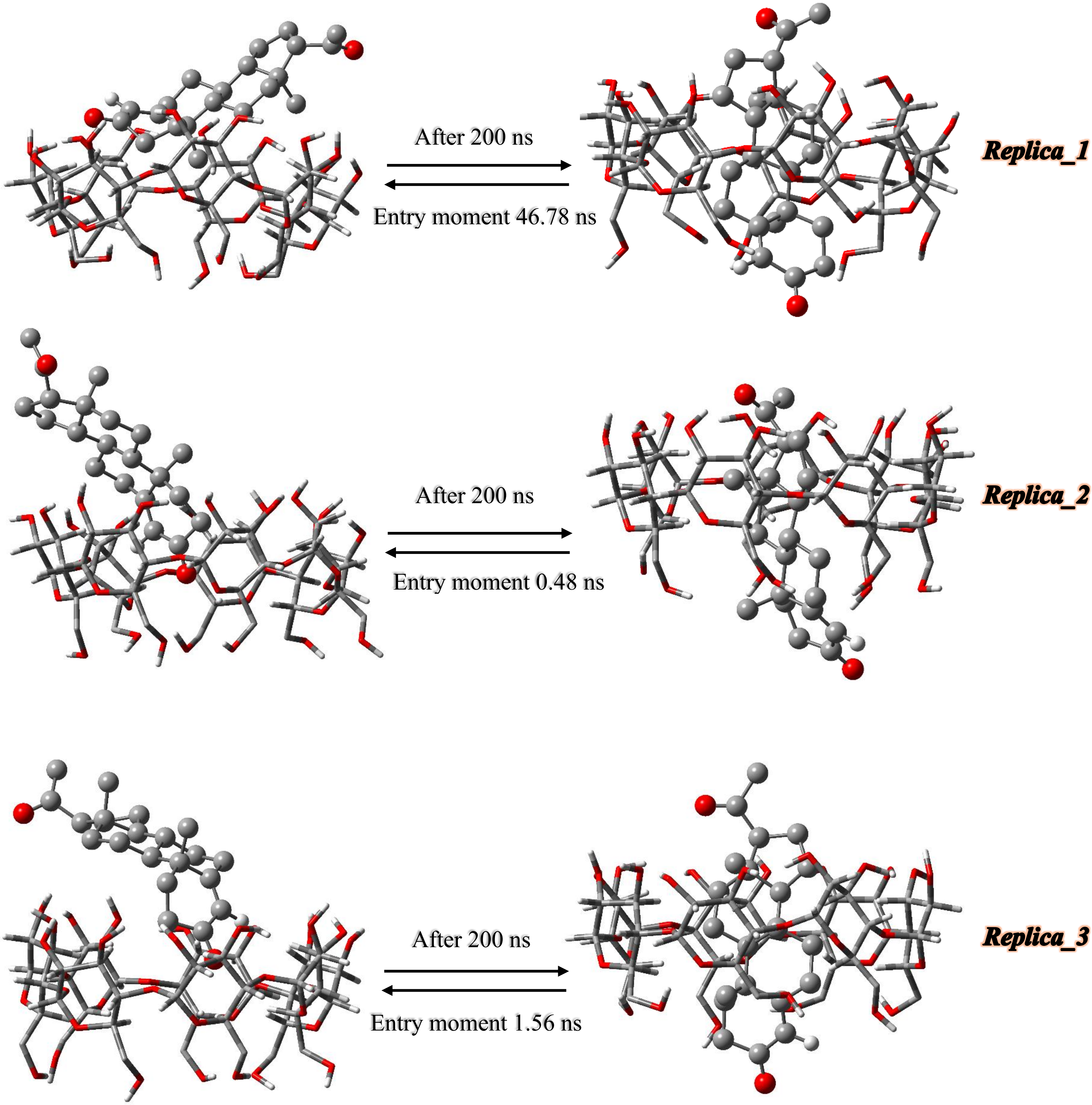
After 200 ns
Entry moment 46.78 ns
Replica_1
After 200 ns
Entry moment 0.48 ns
Replica_2
After 200 ns
Entry moment 1.56 ns
Replica_3

***Continued***

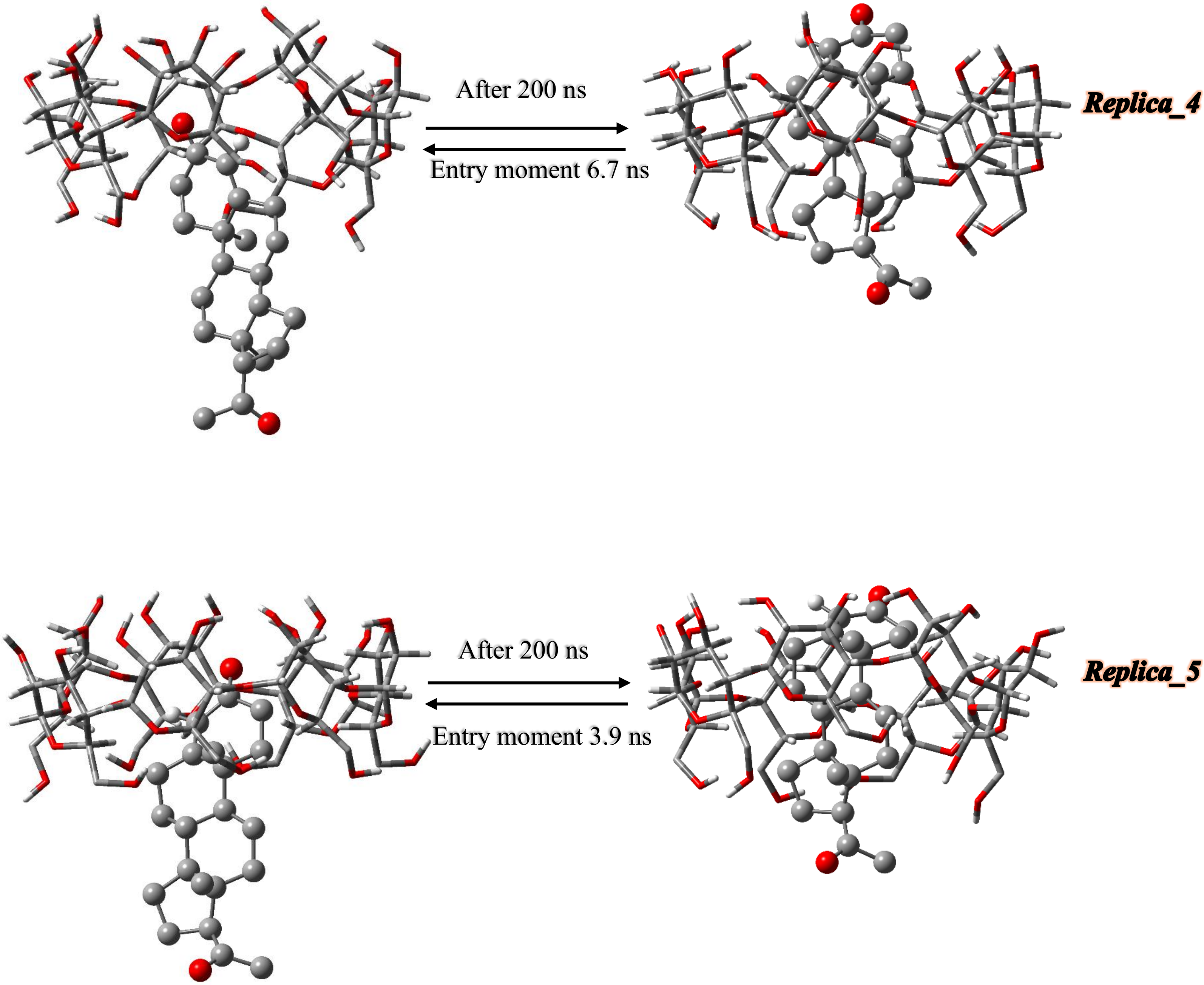


**Figure S4:** Representative snapshots of the moment of entry and loading mechanism of PROG into the βCD cavity (left), along with their final configurations (right) in different replicas.

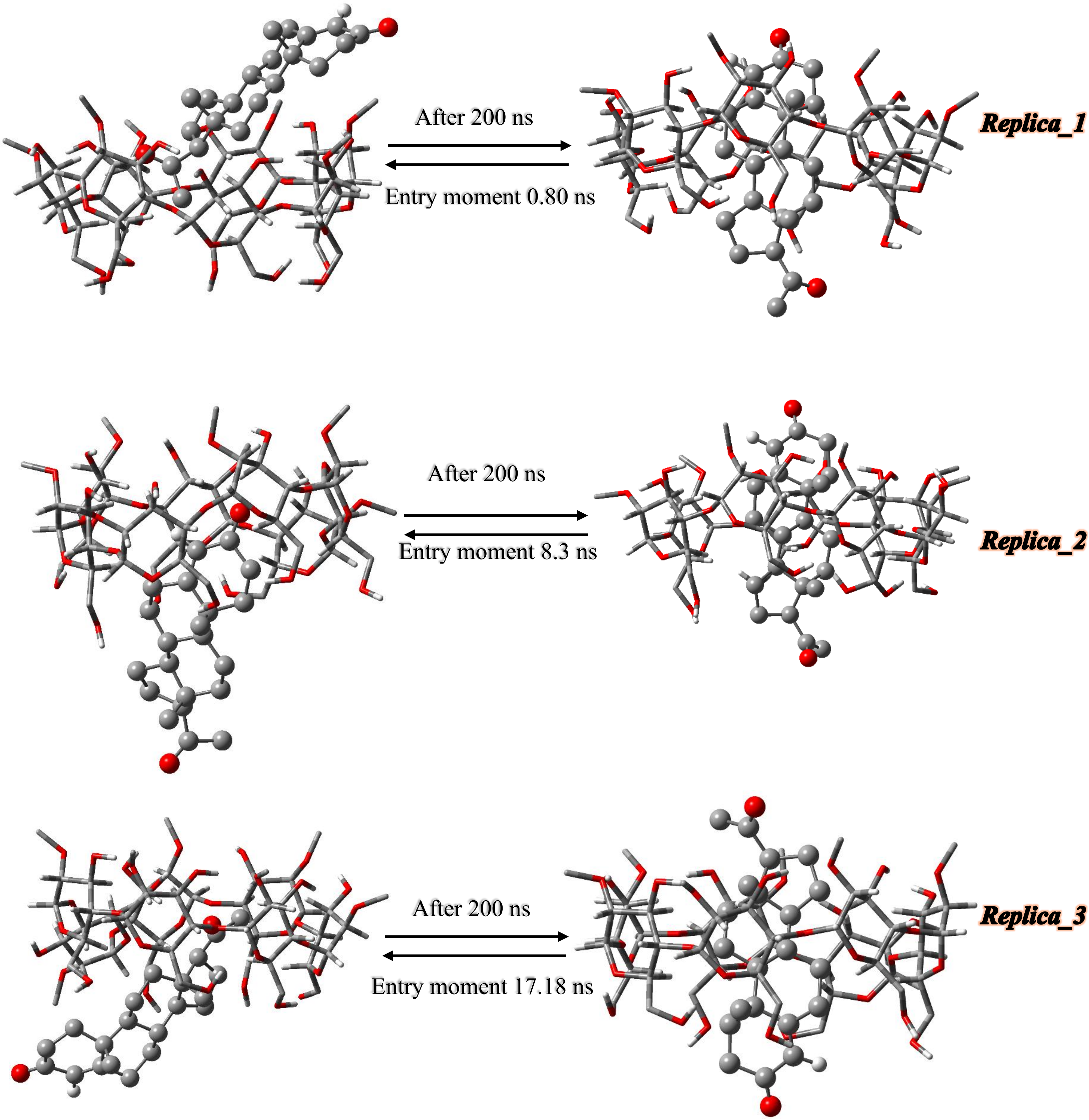
After 200 ns
Entry moment 0.80 ns
Replica_1
After 200 ns
Entry moment 8.3 ns
Replica_2
After 200 ns
Entry moment 17.18 ns
Replica_3

***Continued***

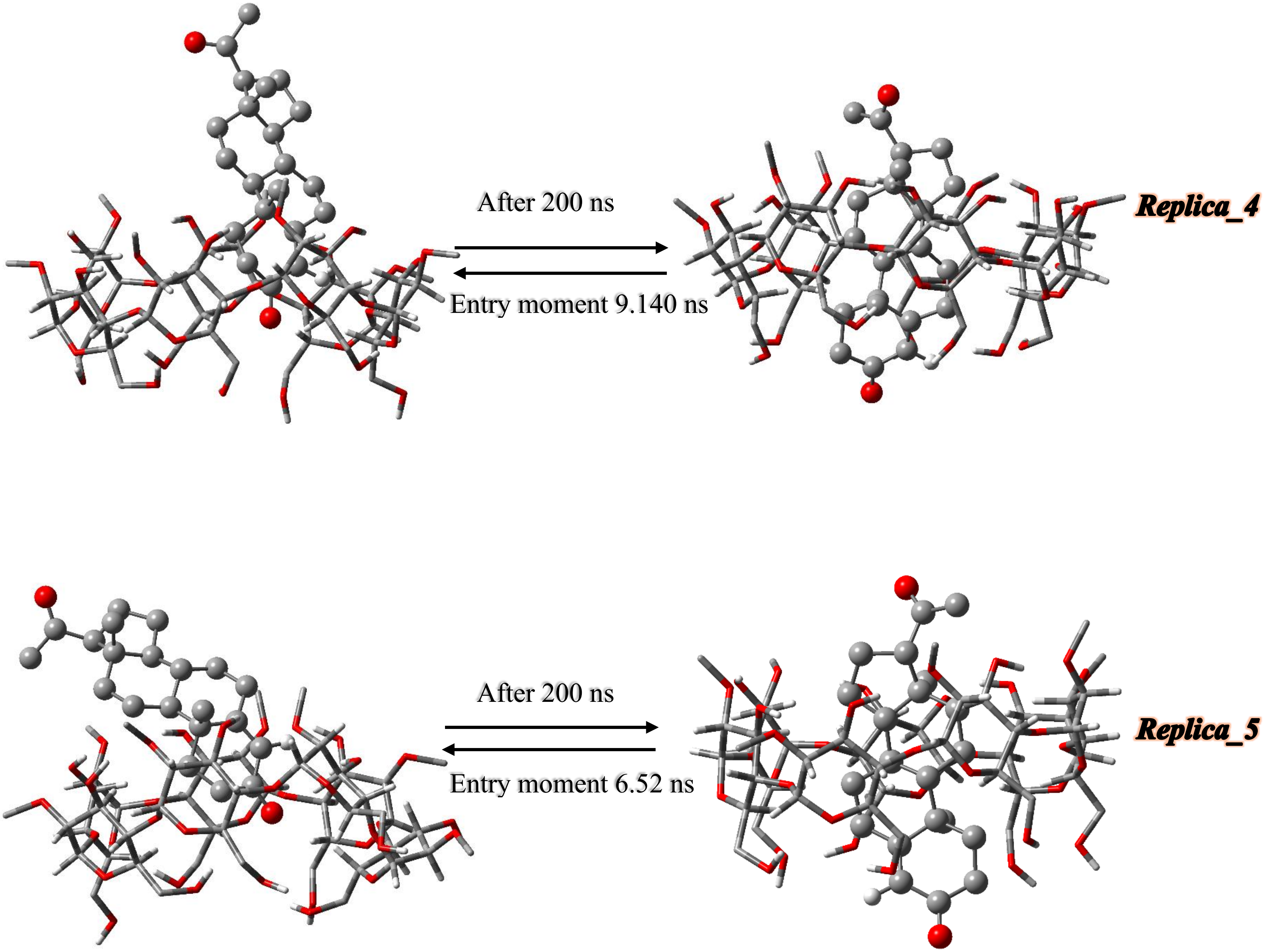


**Figure S5:** Representative snapshots of the moment of entry and loading mechanism of PROG into the 2-MβCD cavity (left), along with their final configurations (right) in different replicas.

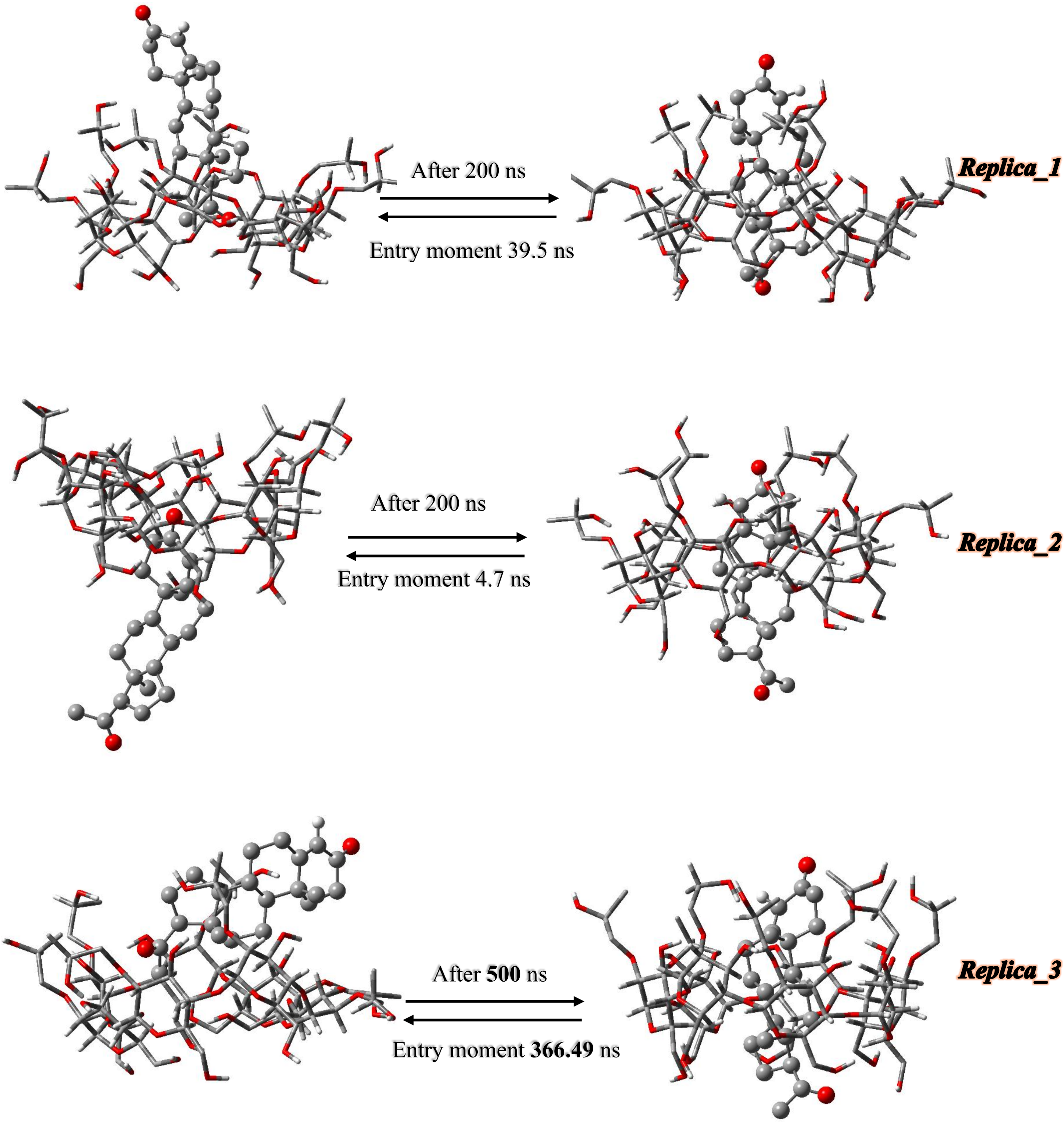
After 200 ns
Entry moment 39.5 ns
Replica_1
After 200 ns
Entry moment 4.7 ns
Replica_2
After **500** ns
Entry moment **366.49** ns
Replica_3

***Continued***

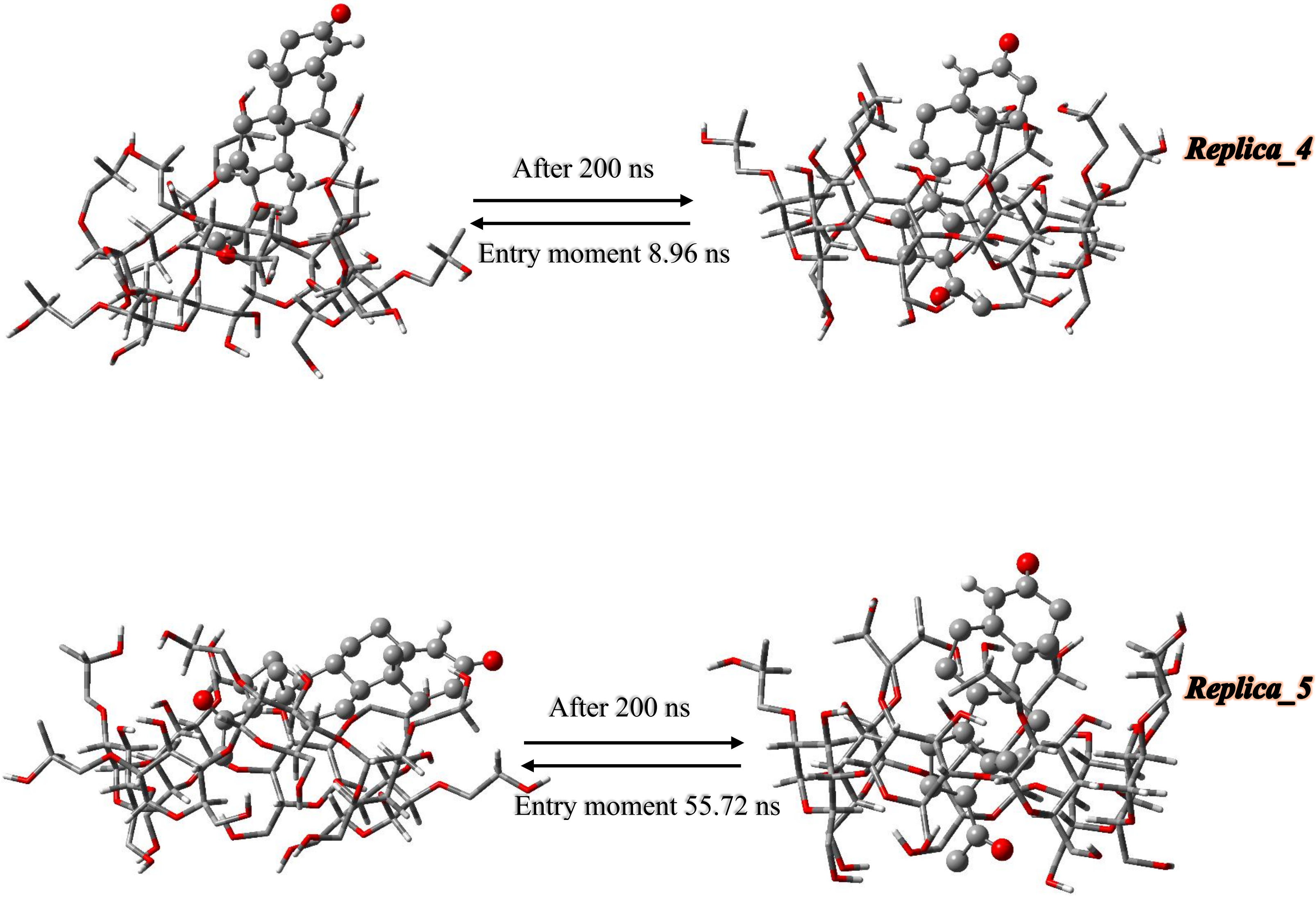


**Figure S6:** Representative snapshots of the moment of entry and loading mechanism of PROG into the 2-HPβCD cavity (left), along with their final configurations (right) in different replicas.

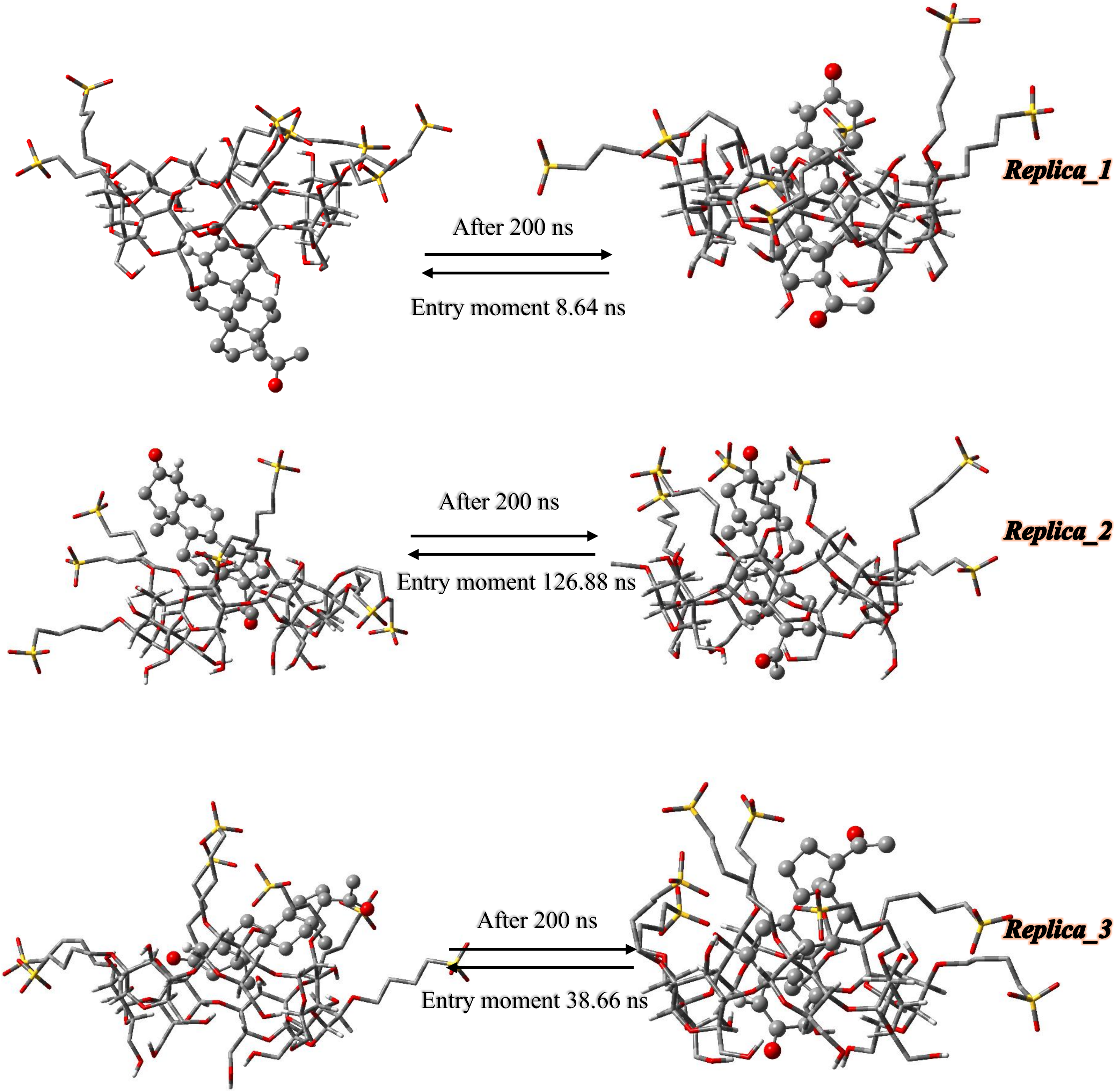
After 200 ns
Entry moment 8.64 ns
Replica_1
After 200 ns
Entry moment 126.88 ns
Replica_2
After 200 ns
Entry moment 38.66 ns
Replica_3

***Continued***

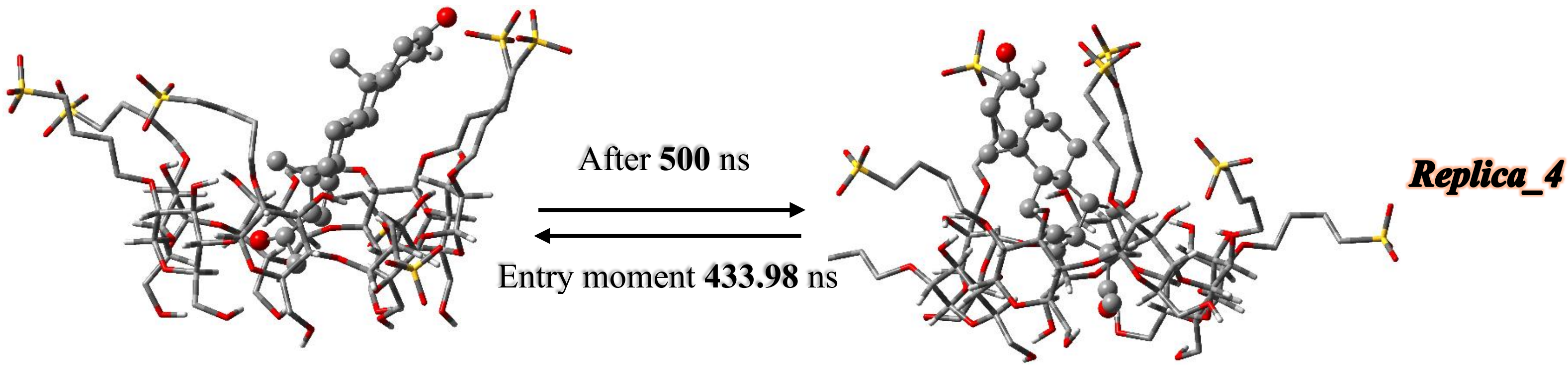


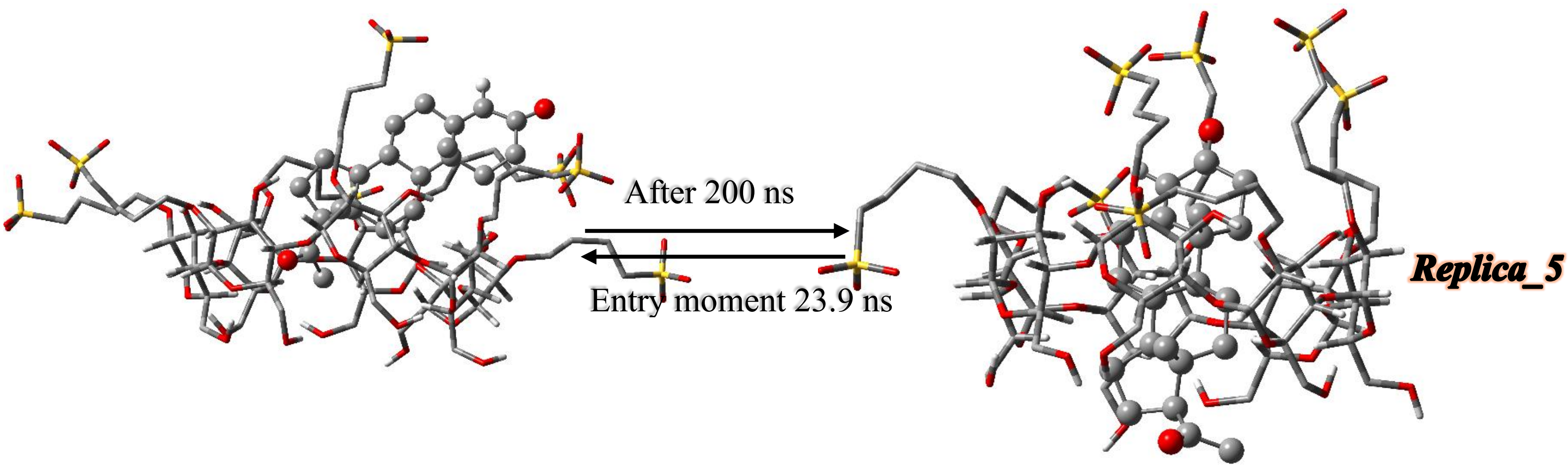


**Figure S7:** Representative snapshots of the moment of entry and loading mechanism of PROG into the 2-SBEβCD cavity (left), along with their final configurations (right) in different replicas.

## Section S3. Additional Structural Characteristics of CDs

### S3.1 Shape descriptor definitions

The gyration tensor contains detailed information on the three-dimensional shape and symmetry properties of molecular structures. Three important characteristics derived from this tensor provide different insights into molecule geometry (spherical, prolate, or oblate). Relative shape anisotropy ($\kappa^2$) quantifies the overall deviation of a molecular structure from perfect spherical symmetry [1,2]. This parameter is particularly significant in pharmaceutical applications since it directly affects the transport capabilities, cellular uptake, and biodistribution of drug delivery systems [3,4]. $\kappa^2$ is calculated using the principal moments of the radius of gyration tensor, which are typically ordered as $\lambda_1 \geq \lambda_2 \geq \lambda_3$. The squared radius of gyration is defined as $R_g^2 = \lambda_1 + \lambda_2 + \lambda_3$, and $\kappa^2$ is then obtained according to the following equation [5]:

$$\kappa^2 = 1 - 3\frac{(\lambda_1\lambda_2 + \lambda_2\lambda_3 + \lambda_1\lambda_3)}{(\lambda_1 + \lambda_2 + \lambda_3)^2} \quad (1)$$

The $\kappa^2$ values range from 0 to 1, where 0 corresponds to a perfectly spherical conformation and 1 represents an ideally linear chain structure.

Asphericity (*b*) measures the degree of deviation from spherical geometry, with particular emphasis on the dominance of the major principal axis. It is calculated as:

$$b = \lambda_1 - \frac{1}{2}(\lambda_2 + \lambda_3) \quad (2)$$

Higher asphericity values indicate more elongated, rod-like structures, while lower values suggest more compact, sphere-like conformations.

Acylindricity (*c*) quantifies the deviation from perfect cylindrical symmetry by measuring the asymmetry between the two minor principal axes [6]:

$$c = (\lambda_2 - \lambda_3) \quad (3)$$

### S3.2 Extended interpretation of shape descriptors

In terms of the interpretation of the indices, a decrease in $\kappa^2$ directly corresponds to an increase in the overall symmetry and a more spherical shape, while b quantifies the degree of "major axis dominance" and c reflects the cross-sectional asymmetry along the two minor axes.

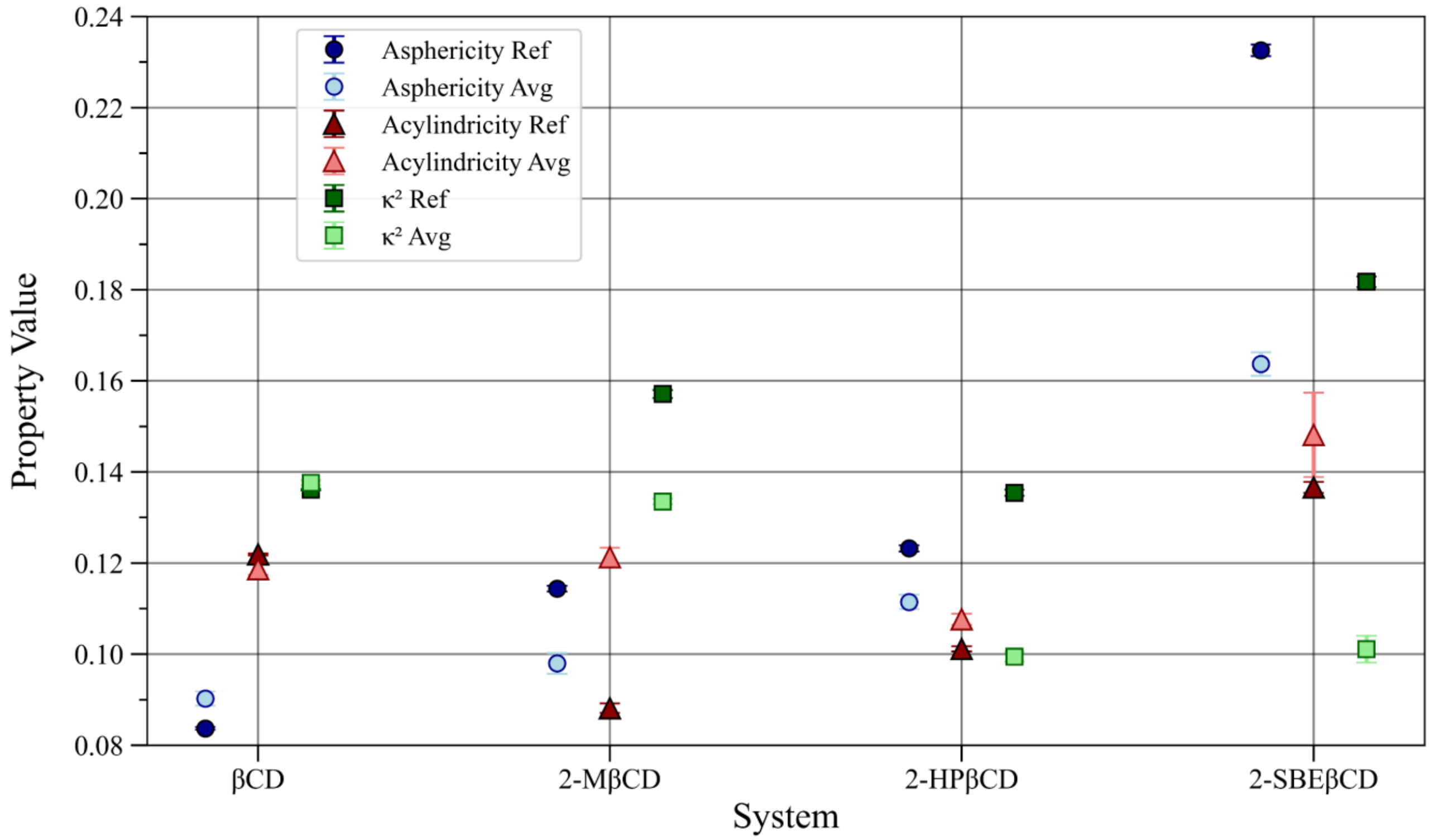


**Figure S8:** Global shape descriptors derived from the gyration tensor ($b$, $c$, $\kappa^2$) for the cyclodextrin systems in water (reference) and in progesterone-bound simulations (replica average). Error bars represent reported uncertainties (reference values) and ±1 standard deviation across replicas.

### S3.3 Extended circularity interpretation

Two mechanistic insights emerge from this trend. First, even after PROG loading, the difference between the $\mathbf{O_2}$ and $\mathbf{O_6}$ rims is maintained; the SHR approaches near-perfect circularity, but the PHR never reaches the same level due to higher flexibility and the effect of the substituents. Second, the magnitude of circularity enhancement correlates directly with substituent bulk and mobility; larger and more flexible substituents cause a greater distortion in the unbound state and therefore exhibit a higher degree of structural correction upon PROG binding, as exemplified by 2-SBEβCD. From a complex stability perspective, the circularization of the rims results in more

uniform peripheral contact and produces a more uniform distribution of rim-atom separations, which may promote more geometrically consistent host–guest contacts.

**S3.4 Extended height interpretation**

From a mechanistic perspective, two points stand out. First, the persistent difference $h_{16} > h_{12}$ even after loading suggests that the primary aperture is inherently more flexible, but in the presence of PROG it rearranges toward a βCD-like height rather than collapsing further. Second, the magnitude of the effect depends on the volume and flexibility of the substituents. The larger and more flexible the substituent, the greater the initial collapse and, consequently, the greater the potential for recovery upon guest binding, explaining why 2-SBEβCD shows the most pronounced height restoration.

**S3.5 Surface-to-volume ratio and moment of inertia**

The larger surface area of nanoparticles relative to their volume facilitates increased interaction with the environment, thereby enabling various applications such as drug delivery and catalysis [7,8]. Comparing the total moment of inertia ($I_{tot}$) and the ratio of the solvent-accessible surface area (SASA) of each CD to its volume (V) with the previously analyzed shape descriptors ($b$, $c$, $\kappa^2$, aperture circularity, and the heights $h_{12}/h_{16}$) yields a coherent structural picture. Itot reports how mass is distributed relative to the center of mass, increasing when sugar units/substituents are displaced outward or when the torus thickens along its principal axis. The S/V ratio quantifies effective solvent exposure relative to overall size and rises when the accessible surface grows (more exposed substituents/smoother rims) and/or the enclosed volume is relatively smaller.

In the PROG-free reference series, $I_{tot}$ increases monotonically with substitution (consistent with added mass and radial extent) whereas S/V decreases relative to βCD, as shown in **Table S1**. This decline parallels reduced h12/h16 and lower circularity, indicating axial compression and rim irregularity that diminish accessible surface per unit volume.

PROG inclusion reverses these trends and drives convergence across derivatives. Replica-averaged Itot shows relative increases of about +0.9% (βCD), +8.8% (2-MβCD), +11.3% (2-HPβCD), and +5.6% (2-SBEβCD) versus reference. These gains align with restored axial thickness (larger $h_{12}$ and $h_{16}$), more circular apertures, and reduced $\kappa^2$ (greater global symmetry): mass shifts slightly outward but more uniformly, raising Itot. S/V likewise increases by ~+0.5%, +3.8%, +7.2%, and +7.0% in the same order and collapses to a narrow band across all derivatives, mirroring the convergence seen in circularity and heights. Geometrically, PROG acts as an internal

mold that fills the cavity, enforces continuous wall contact, and lifts substituents from recessed to exposed configurations, smoothing rims and enlarging accessible surface more than volume.

The magnitude of these responses correlates with substituent bulk and conformational freedom: 2-HPβCD exhibits the largest relative rise in $I_{tot}$, followed by 2-MβCD and 2-SBEβCD, while βCD remains essentially unchanged; in S/V, the bulkier HP and especially SBE derivatives show the largest boosts (≈7%).

**Table S1:** The surface-to-volume ratio (S/V) and moment of inertia ($I_{tot}$) of carriers in different simulated systems[a].

| Properties / Systems | S/V ($nm^{-1}$) | I (a.m.u×$nm^2$) |
|---|---|---|
| βCD + Water | 4.873 ± 0.002 | 496.06 ± 0.3 |
| βCD + Water + PROG | 4.897 ± 0.002 | 500.51 ± 0.17 |
| 2-MeβCD + Water | 4.733 ± 0.005 | 532.82 ± 12.70 |
| 2-MeβCD + Water + PROG | 4.912 ± 0.004 | 579.94 ± 0.29 |
| 2-HPβCD + Water | 4.514 ± 0.003 | 762.34 ± 5.78 |
| 2-HPβCD + Water + PROG | 4.839 ± 0.002 | 848.68 ± 1.91 |
| 2-SBEβCD + Water | 4.623 ± 0.003 | 1558.74 ± 19.73 |
| 2-SBEβCD + Water + PROG | 4.948 ± 0.005 | 1646.08 ± 10.36 |

[a]All results were obtained from the last 10 % of the simulation time. The result for PROG-containing systems is an average of five replicas. the uncertainty was obtained by propagation of the statistical errors of SASA and volume estimated from block averaging.

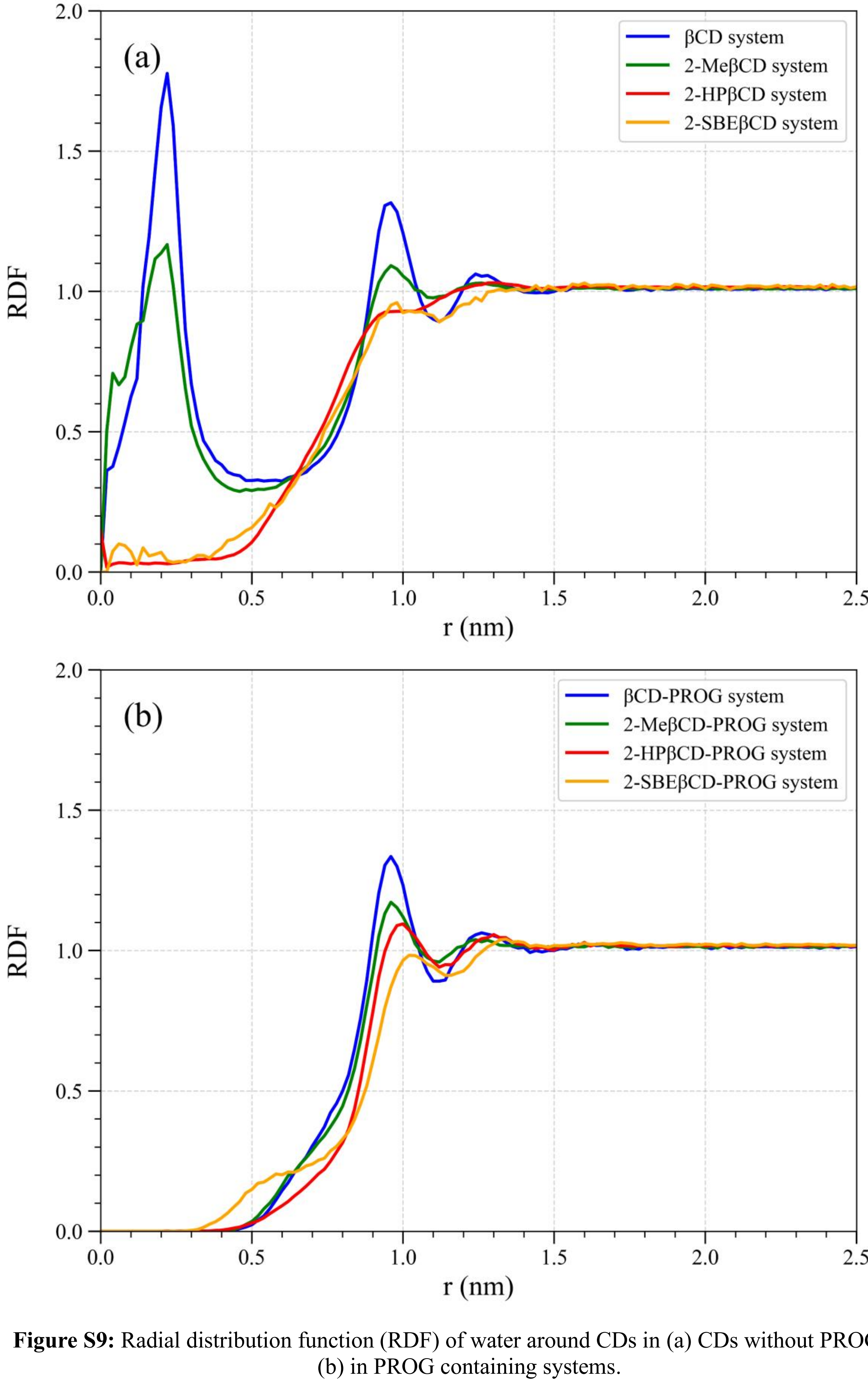


**Figure S9:** Radial distribution function (RDF) of water around CDs in (a) CDs without PROG, and (b) in PROG containing systems.

**Section S4. Tetrahedral Order Parameter: Definition and Interpretation**

The tetrahedral order parameter (q) characterizing the local structure of a chosen water molecule, based on the orientation of its four nearest neighbors, is defined [9] as:

$$q = 1 - \frac{3}{8}\sum_{j=1}^{3}\sum_{k=j+1}^{4}(cos\psi_{jk} + \frac{1}{3})^2 \tag{4}$$

where $\psi_{jk}$ is the angle formed by neighbors $j$ and $k$ with the central molecule $i$, and the summation involves six such angles. The possible range for the tetrahedral order parameter of a molecule is $-3 \leq q \leq 1$. The normalization factor of $\frac{3}{8}$ in eq. 4, however, ensures that the ensemble averaged tetrahedral order parameter, $\langle q \rangle$, spans the range $0 \leq \langle q \rangle \leq 1$. The value of 1 corresponds to a perfect tetrahedral arrangement of neighboring molecules about the central molecule, as in the ideal tetrahedral structure of ice, while the value of 0 indicates a random mutual arrangement of molecules as in an ideal gas [10].

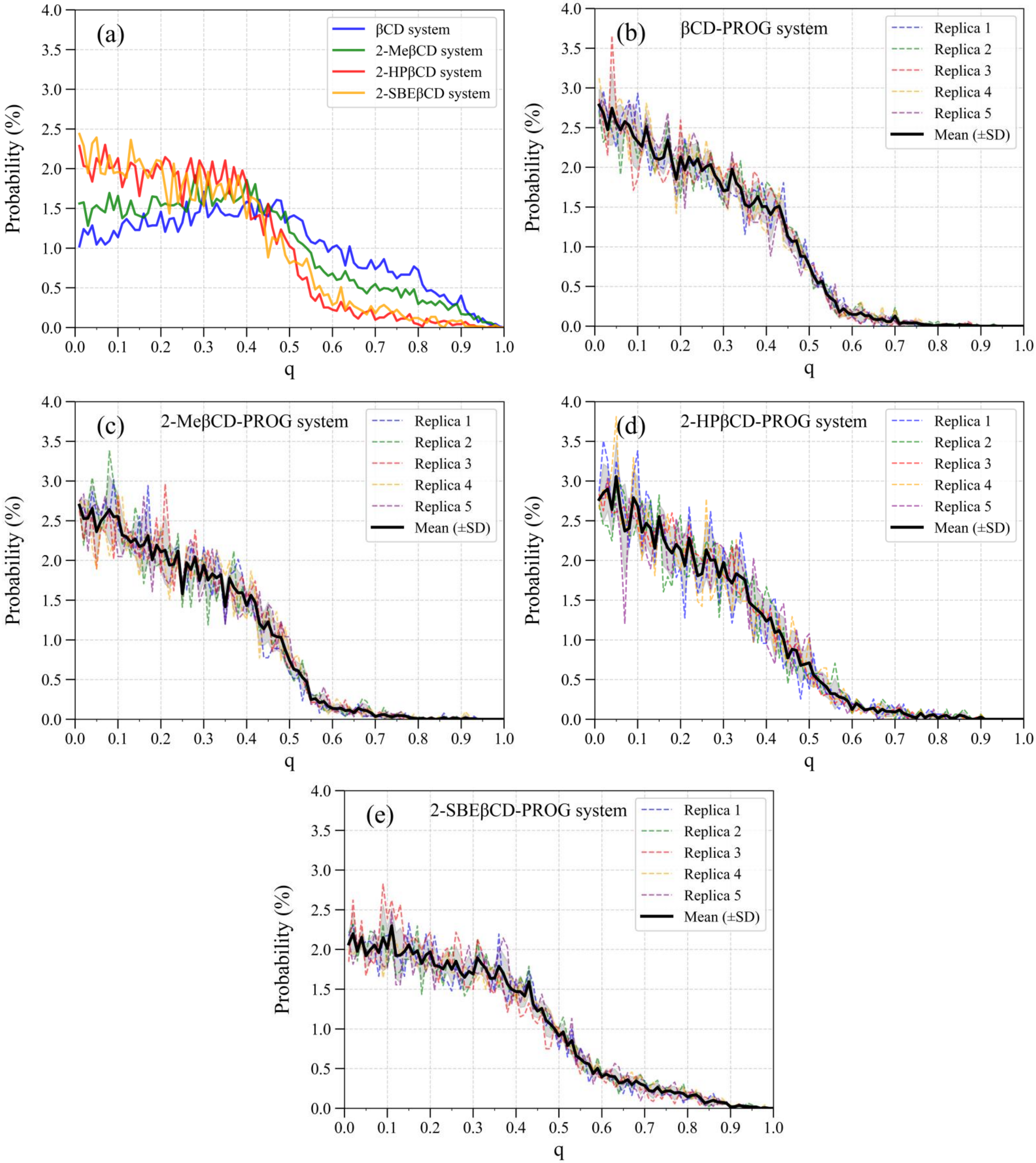


**Figure S10:** Tetrahedral order parameter (q) of water molecules within r < 0.8 nm from the cavity center of CDs (a) CDs without PROG, (b) different replicas of the βCD–PROG system, (c) different replicas of the 2-MeβCD–PROG system, (d) different replicas of the 2-HPβCD–PROG system, and (e) different replicas of the 2-SBEβCD–PROG system.

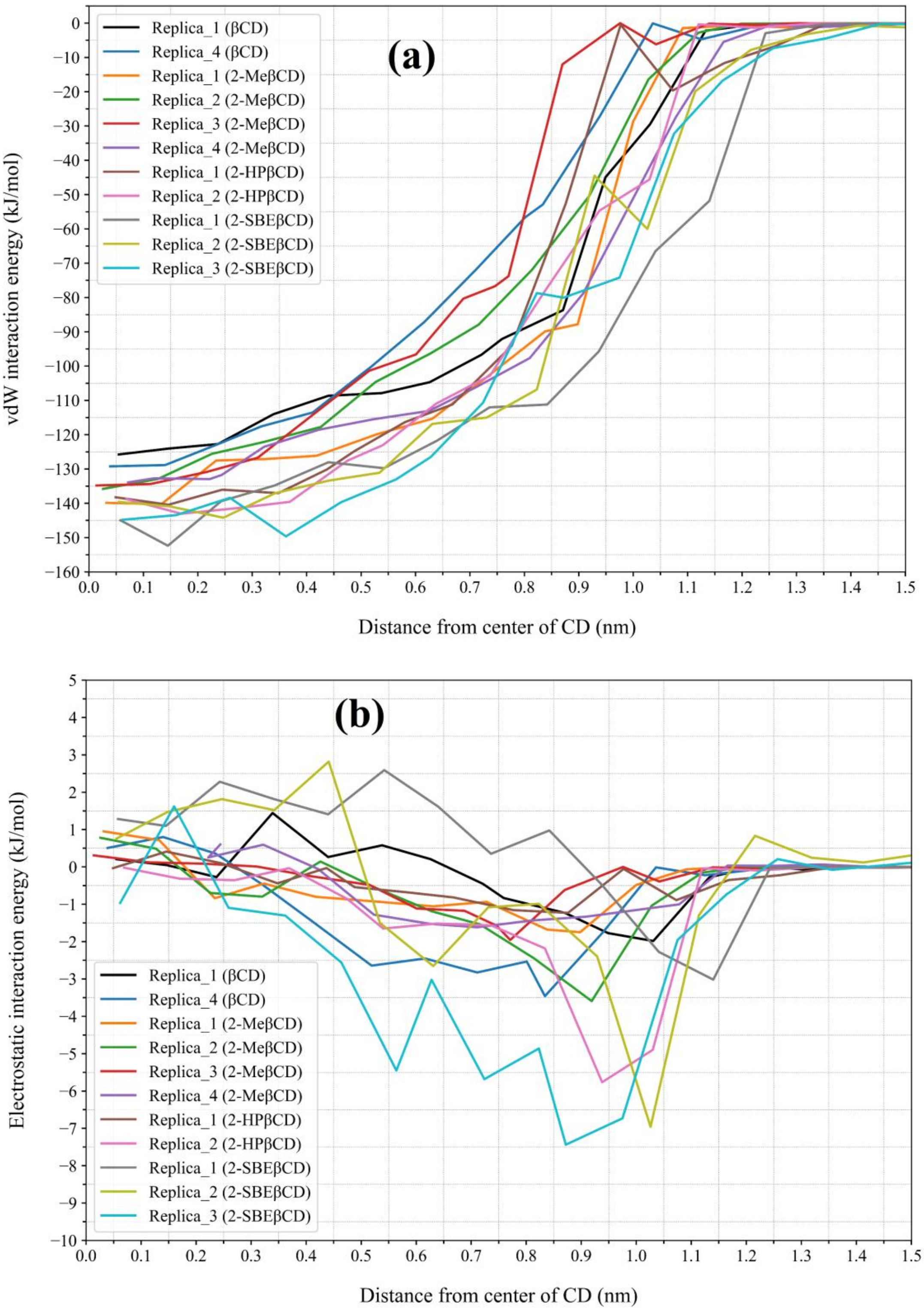


**Figure S11:** Short-range non-bonded (a) van der Waals (vdW) and (b) Coulombic interaction energies between PROG and the CDs as a function of the distance from the CD cavity center.

**Table S2:** Decomposition of sort-range non-bonded interaction energies (van der Waals and Coulomb) between progesterone–cyclodextrin (PROG–CD) complexes and water molecules. Values are reported separately for each replica across the different cyclodextrin systems.

| System \ Energy contribution | vdW (kJ/mol) | Coulombic (kJ/mol) |
|---|---|---|
| βCD (Replica 1) | -277.75 (±0.62) | -260.09 (±0.97) |
| βCD (Replica 2) | -277.42 (±0.90) | -262.73 (±1.00) |
| βCD (Replica 3) | -277.11 (±0.64) | -263.77 (±1.70) |
| βCD (Replica 4) | -275.95 (±0.17) | -260.18 (±1.30) |
| βCD (Replica 5) | -275.11 (±0.23) | -260.99 (±0.77) |
| 2-MβCD (Replica 1) | -311.18 (±0.23) | -233.52 (±1.40) |
| 2-MβCD (Replica 2) | -311.83 (±0.64) | -233.77 (±1.10) |
| 2-MβCD (Replica 3) | -315.24 (±0.75) | -235.49 (±0.98) |
| 2-MβCD (Replica 4) | -314.85 (±0.30) | -236.06 (±1.10) |
| 2-MβCD (Replica 5) | -314.32 (±1.40) | -236.09 (±0.89) |
| 2-HPβCD (Replica 1) | -364.75 (±1.10) | -268.94 (±1.80) |
| 2-HPβCD (Replica 2) | -365.20 (±1.40) | -270.30 (±1.50) |
| 2-HPβCD (Replica 3) | -363.32 (±0.45) | -275.89 (±0.85) |
| 2-HPβCD (Replica 4) | -365.43 (±1.60) | -271.12 (±1.30) |
| 2-HPβCD (Replica 5) | -363.41 (±1.10) | -270.75 (±0.91) |
| 2-SBEβCD (Replica 1) | -133.33 (±1.20) | -2864.61 (±11.00) |
| 2-SBEβCD (Replica 2) | -133.71 (±1.50) | -2855.61 (±5.20) |
| 2-SBEβCD (Replica 3) | -125.25 (±2.30) | -2863.25 (±2.80) |
| 2-SBEβCD (Replica 4) | -131.13 (±1.30) | -2874.64 (±4.20) |
| 2-SBEβCD (Replica 5) | -131.54 (±2.70) | -2877.64 (±10.00) |

**References**:


[1] V. Blavatska, W. Janke, Polymer adsorption on a fractal substrate: Numerical study, J. Chem. Phys. 136 (2012) 104907.

[2] V. Blavatska, W. Janke, Shape anisotropy of polymers in disordered environment, J. Chem. Phys. 133 (2010) 184903.

[3] J.A. Champion, S. Mitragotri, Role of target geometry in phagocytosis, Proceedings of the National Academy of Sciences 103 (2006) 4930–4934.

[4] E.A. Simone, T.D. Dziubla, V.R. Muzykantov, Polymeric carriers: role of geometry in drug delivery, Expert Opin. Drug Deliv. 5 (2008) 1283–1300.

[5] H. Arkın, W. Janke, Gyration tensor based analysis of the shapes of polymer chains in an attractive spherical cage, J. Chem. Phys. 138 (2013) 54904.

[6] J. Vymětal, J. Vondrášek, Gyration-and inertia-tensor-based collective coordinates for metadynamics. Application on the conformational behavior of polyalanine peptides and Trp-cage folding, J. Phys. Chem. A 115 (2011) 11455–11465.

[7] A. Banerjee, J. Qi, R. Gogoi, J. Wong, S. Mitragotri, Role of nanoparticle size, shape and surface chemistry in oral drug delivery, Journal of Controlled Release 238 (2016) 176–185.

[8] D. Astruc, Introduction: nanoparticles in catalysis, Chem. Rev. 120 (2020) 461–463.

[9] J.R. Errington, P.G. Debenedetti, Relationship between structural order and the anomalies of liquid water, Nature 409 (2001) 318–321.

[10] E. Duboué-Dijon, D. Laage, Characterization of the local structure in liquid water by various order parameters, J. Phys. Chem. B 119 (2015) 8406–8418.
